# Spatial, Temporal, and Density-Dependent Components of Habitat Quality for a Desert Owl

**DOI:** 10.1371/journal.pone.0119986

**Published:** 2015-03-18

**Authors:** Aaron D. Flesch, Richard L. Hutto, Willem J. D. van Leeuwen, Kyle Hartfield, Sky Jacobs

**Affiliations:** 1 School of Natural Resources and the Environment, University of Arizona, Tucson, Arizona, United States of America; 2 Division of Biological Sciences, University of Montana, Missoula, Montana, United States of America; 3 Watershed Management Group, Tucson, Arizona, United States of America

## Abstract

Spatial variation in resources is a fundamental driver of habitat quality but the realized value of resources at any point in space may depend on the effects of conspecifics and stochastic factors, such as weather, which vary through time. We evaluated the relative and combined effects of habitat resources, weather, and conspecifics on habitat quality for ferruginous pygmy-owls (*Glaucidium brasilianum*) in the Sonoran Desert of northwest Mexico by monitoring reproductive output and conspecific abundance over 10 years in and around 107 territory patches. Variation in reproductive output was much greater across space than time, and although habitat resources explained a much greater proportion of that variation (0.70) than weather (0.17) or conspecifics (0.13), evidence for interactions among each of these components of the environment was strong. Relative to habitat that was persistently low in quality, high-quality habitat buffered the negative effects of conspecifics and amplified the benefits of favorable weather, but did not buffer the disadvantages of harsh weather. Moreover, the positive effects of favorable weather at low conspecific densities were offset by intraspecific competition at high densities. Although realized habitat quality declined with increasing conspecific density suggesting interference mechanisms associated with an Ideal Free Distribution, broad spatial heterogeneity in habitat quality persisted. Factors linked to food resources had positive effects on reproductive output but only where nest cavities were sufficiently abundant to mitigate the negative effects of heterospecific enemies. Annual precipitation and brooding-season temperature had strong multiplicative effects on reproductive output, which declined at increasing rates as drought and temperature increased, reflecting conditions predicted to become more frequent with climate change. Because the collective environment influences habitat quality in complex ways, integrated approaches that consider habitat resources, stochastic factors, and conspecifics are necessary to accurately assess habitat quality.

## Introduction

Understanding how the environment affects the fitness realized by individuals is a fundamental aspect of ecology. Environmental factors that vary in space and time influence habitat quality by affecting the fitness realized by occupants in a given habitat. Fitness is often defined as an individual’s contribution to population growth [1,2]. Accordingly, habitat quality or habitat fitness potential (*sensu* [3]) can be defined as the contribution of individuals in a specific habitat to population growth over periods that exceed the generation time of the focal species [4–6]. Ultimately, habitat quality should affect settlement choices by individuals because those choices have important demographic consequences and are under natural selection [7]. Thus, understanding factors that influence habitat quality can elucidate important selective pressures and guide management.

Environmental factors that drive habitat quality can be organized into a spatial and temporal component and a component related to the effects of conspecifics. Spatial factors are those that vary across space at any given point in time, often in predictable ways from the perspective of a focal organism. Temporal factors in contrast, vary with time at any given point in space sometimes in unpredictable ways. The effect of conspecifics varies spatially and temporally and is considered separately because conspecifics affect the realized value of resources that may otherwise be of high intrinsic value. Although factors associated with each component vary to some extent in space and time, a framework with these components provides a useful context for assessing how the environment affects habitat quality, which is a goal of this study.

Factors associated with the spatial component of habitat quality are often described collectively as habitat, which is a set of resources and conditions that foster occupancy and persistence of individuals of a given species across time [8]. This definition of habitat is similar to that of the niche [9] but represents a projection or mapping of the niche in space. Although environments of similar structure and physiognomy are often defined as the same habitat [10], different places even within similar environments can drive differences in individual performance (e.g. fitness realized by an individual) due to variation in resources they provide. Regardless of the specific resources that comprise habitat, their functional roles in providing food and reducing vulnerability to physiological stress and heterospecific enemies are fundamental [11]. Although efforts to identify factors that affect habitat quality are common, until recently, most have focused on indirect measures such as body condition, settlement timing, or density, rather than on direct estimates of vital rates [6]. In systems where vital rates have been monitored over time, spatial variation in vegetation, landscape structure, and abiotic factors have been found to have large and consistent effects on performance that persist longer than the generation time of the focal species [5,12–15]. Thus, in some systems, good places tend to remain good for long periods.

Factors associated with the temporal component of habitat quality are related to both deterministic (e.g., seasonality) and stochastic fluctuations in environmental conditions that affect vital rates and thus population dynamics [16,17]. Although such stochastic fluctuations are sometimes considered random noise, temporal variation in weather can have large effects on performance through either direct (physiological) or indirect (food web) pathways despite unpredictable timing [18]. Thus, even though spatial factors such as vegetation structure may be the primary cues used by animals to choose high-quality habitat [10], future conditions normally associated with those cues may not be realized due to unpredictable weather, which if extreme can produce major ecological crunches or bonanzas [19]. Thus, realized habitat quality at a given point in time may be poor even at points in space that tend to be good over time. Moreover, the combined effects of weather and spatial variation in resources on performance can act in an additive or interactive manner. If weather effects are additive, they will be uniform across space and habitat may not attain its full potential until conditions are favorable. If weather effects are interactive, some resources may buffer the negative effects of harsh weather [5,19] or amplify the benefits of favorable weather, which has important implications for management in the face of climate change.

The abundance of conspecifics occupying a focal area is another important component of the environment that can affect performance [20,21]. Individuals in habitat of high fundamental quality (e.g., basic suitability *sensu* [22], zero-density suitability *sensu* [23], or intrinsic habitat value *sensu* [15]), for example, may not realize the potential of that habitat due to intraspecific competition. At one extreme, under the Ideal Free Distribution (IFD), intraspecific competition equalizes realized habitat quality among individuals despite differences in fundamental quality of habitat they occupy [24]. Mounting antagonistic interactions and reductions in territory size likely drive these patterns [25–27]. At the opposite extreme, under the Ideal Despotic Distribution (IDD), individual competitive abilities vary, and dominants relegate subordinates to habitat of lower quality and thereby realize better performance [24]. Although often viewed as alternatives, processes that drive each distribution may operate simultaneously on the same or different vital rates [28,29] just as they do on feeding rates [30], and create a continuum of potential responses to conspecifics [31]. Moreover, whereas realized habitat quality may or may not decline with conspecific density, magnitudes of density dependence may depend on fundamental habitat quality [32,33]. Thus, in systems that conform strictly to the IDD, spatial variation in resources alone explain habitat quality whereas in systems with properties of both distributions, realized habitat quality will vary spatially and decline with conspecific density either uniformly in all habitat types or at rates that depend on fundamental habitat quality.

When the combined effects of each environmental component are integrated, other potential explanations of habitat quality emerge. Harsh weather, for example, could depress performance more when conspecific densities are high [34,35], or the benefits of favorable weather could be offset by competition. Moreover, the combined effects of weather and conspecifics could be more complex if they also depend on habitat resources.

Although the effects of factors associated with each component of habitat quality have been well studied individually, very few studies have assessed the combined effects of habitat, weather, and conspecifics on the performance of individuals in wild animal populations. Thus, our understanding of how the collective environment influences habitat quality is incomplete, especially across continuous variation in important resources that drive fundamental habitat quality. The most problematic aspects of existing studies include the following: (1) they rarely consider how variation in resources and conspecifics affect performance at individual vs. population scales [11,36], (2) they often define habitat as discrete entities [22,37] that may not exist in the eyes of focal organisms, and (3) they consider time periods too short to capture sufficient variation in temporal factors. With respect to the last issue, inferences on the effects of habitat resources could be misleading if they fail to consider the broader temporal context, which may include large effects of weather and conspecifics [19,37]. With respect to the second issue, classifying habitat into discrete types is useful for developing theory, but fails to consider the fact that habitat is often comprised of an intricate combination of many resources that vary continuously in space and time [5,38], and that variation in important resources at microhabitat and territory-specific scales may be more important than that at larger macrohabitat scales (e.g., woodland vs. shrubland).

In addition to environmental components, intrinsic factors related to an individual’s ability to cope with the environment can also affect performance. Age and experience, for example, can affect performance independent of resources [13,39] and maternal effects due to genetics or the environment can affect individual quality [40]. Thus, habitat fitness potential may be driven by a combination of intrinsic and environmental factors, which could interact, or the fitness potential of an individual may not be realized until optimal habitat is occupied. Nonetheless, individual effects are often small relative to environmental ones [6,41–43] and can dissipate over time [44]. Moreover, because the best individuals often have access to the best habitat, intrinsic factors tend to be highly correlated with external factors that affect performance [45–47]. Thus, while we recognize intrinsic differences among individuals exist, they are not considered further because our goal is to understand how the relative quality of different points in space varies across time for the average individual.

We assessed the independent and integrated effects of habitat resources, weather, and conspecifics on habitat quality for a Neotropical owl based on 10 years of monitoring across broad gradients in each environmental component. First, we assessed the extent to which performance varied across space and time. Second, we identified specific factors that explained performance by evaluating hypotheses associated with each component. Third, we assessed the relative importance of components by estimating quantities of variation in performance they explained. Finally, we assessed the integrated effects of all components by evaluating evidence for additive and interactive relationships among important factors associated with each component.

## Materials and Methods

### Study system

**Fig 1:**
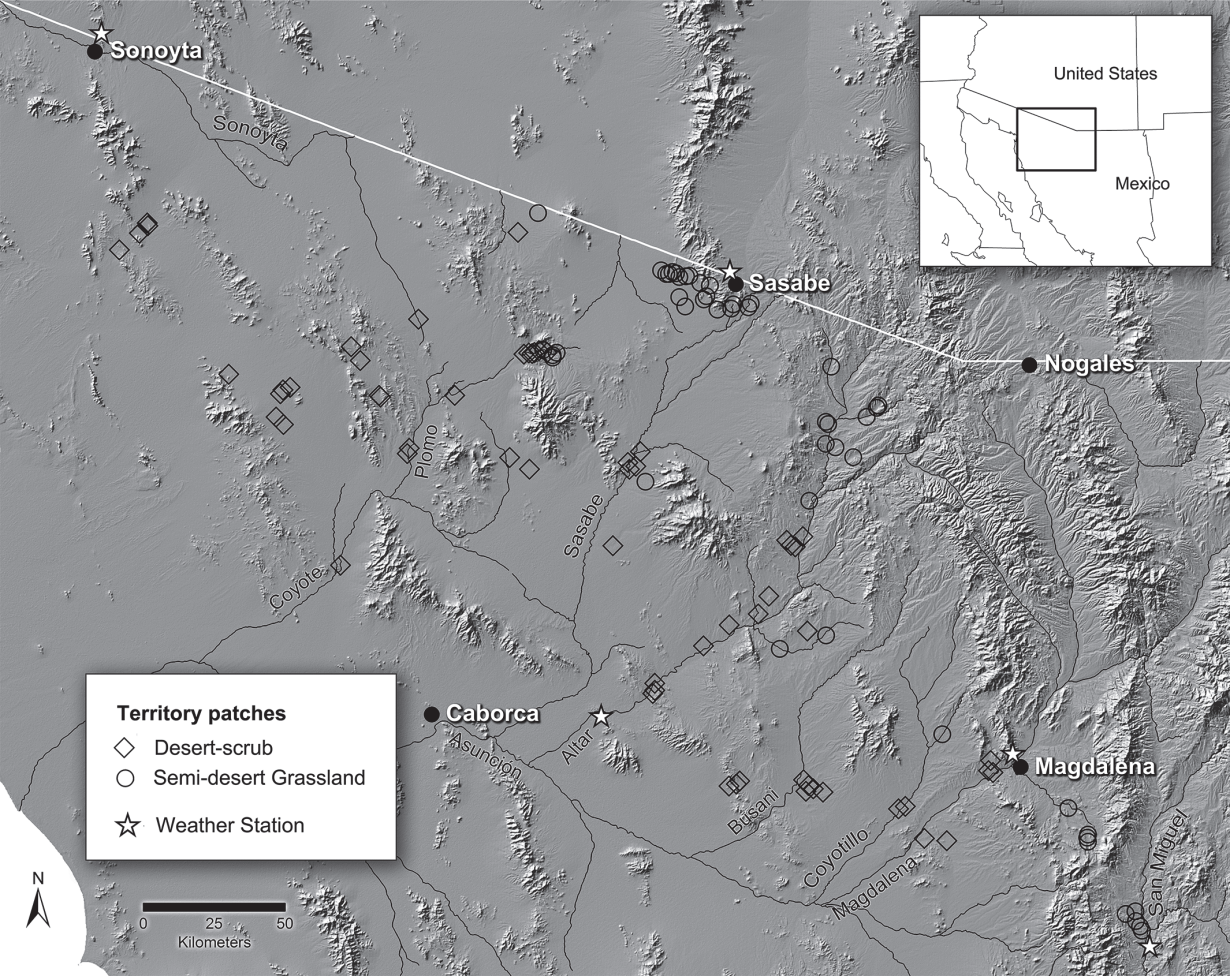
Study area in northwest Mexico showing distribution of territory patches used by ferruginous pygmy-owls and the location of weather stations. Territory patches were located in two major vegetation communities and weather stations were located near Sasabe, Sonoyta, Cucurpe, Magdelena, and Altar. Regional patch occupancy was estimated in 11 regions: San Miguel, upper Magdalena, Magdalena-Coyotillo, Busani, upper Alter, lower Altar, upper Sasabe, lower Sasabe, upper Plomo, lower Plomo, and Sonoyta. Territory patches were 50 ha in area and are not shown to scale; the study area was approximately 20,000 km^2^.

We studied ferruginous pygmy-owls (*Glaucidium brasilianum*) in the Sonoran Desert of northwest Mexico directly south of Arizona, USA (Fig. 1). Pygmy-owls are residents of the lowland Neotropics north to Arizona. Although once common locally in southern Arizona, pygmy-owls were extirpated from much of their range due to habitat loss [48]. As a result, they were listed as endangered in Arizona in 1997 but delisted in 2006 for reasons unrelated to recovery [49]. In nearby Mexico, pygmy-owls are more common, use similar environments, and are declining [50]. Mexican populations are important for recovery in Arizona because dispersal from Mexico can augment populations, especially when combined with habitat restoration.

Pygmy-owls are territorial, raise one brood per year, and exhibit high variation in annual reproductive output (0–6) in the region. In the Sonoran Desert, pygmy-owls mainly consume lizards, and secondarily insects, birds, and mammals, and habitat is confined largely to riparian woodlands of mesquite (*Prosopis velutina*) and other microphyllous trees, and to adjacent upland desert-scrub and semi-desert grassland with giant saguaro cacti (*Carnegiea gigantea*), which provide nest cavities. Desert-scrub is composed of woodland and scrub of short trees such as mesquite, shrubs such as creosote (*Larrea tridentata*) and bursage (*Ambrosia* sp.), and cacti. Semi-desert grassland is composed of savannah and open woodland of mesquite, grasses, and sub-shrubs. Climate is arid to semi-arid with precipitation focused during a summer monsoon and winter storms of Pacific origin. Summers are hot with maximum temperatures >40°C and winters are cool with minimum temperatures near 0°C. Pygmy-owls establish breeding territories in Jan-Mar, lay eggs in Apr, and brood in May-June.

In Arizona, pygmy-owls were the focus of a major controversy between developers and conservationists in the 1990s. At that time, pygmy-owls occupied lands with high economic and conservation value near Tucson. Although controversy subsided with delisting and recent extirpation of pygmy-owls near Tucson, the owl remains a focal species for conservation. Current questions with important implications include understanding factors that affect habitat quality, importance of riparian woodland, and effects of anthropogenic disturbance.

### Design

The basic unit of our study are individual territory patches that can each be occupied by single territorial individuals or breeding pairs. This approach is advantageous because differences in resources and conditions at this scale should be closely linked to variation in individual performance [51], and because variation in individual quality of different animals that occupy patches averages out over time and is thus less likely to be influential [44,52]. In 2001 and 2002, we randomly selected survey transects across the study area, surveyed owls by broadcasting territorial calls in a manner that yields nearly perfect detection probability [53], searched for nests along occupied transects and in other areas selected opportunistically, and located the nests of most individuals. In subsequent years through 2010, we surveyed areas around nests (or owl locations if nests were not found initially) from prior years and found nearly all nests.

We defined territory patches based on recurring patterns of use by owls by plotting nest coordinates across time and delineating clusters of use in space. Although owls nested in different cavities in some years, mean within-patch distances between nests in successive years ($\overset{-}{x\;}$± SE = 226 ± 13 m) was 5.5 times lower than that between nests in neighboring patches. Thus, because we located the nests of most owls and because the distribution of potential nests was clumped, this approach allowed easy identification of patches. To represent patches, we placed 50-ha circles around the average coordinates of all nests within each patch, which minimized overlap with neighboring patches, included 98% of nests associated with patches, and is similar in area to breeding home ranges.

Habitat quality represents individual contributions to population growth from a specific habitat, and thus, is a function of both reproduction and survival. At the scale of individual territory patches, however, challenges in gathering sufficient demographic data have precluded estimating territory-specific population growth rates (λ_h_) with few exceptions [5,54]. We used territory-specific estimates of reproductive output (R) measured each year over 10 years to index habitat quality. This approach is reasonable because in many vertebrates spatiotemporal variation in adult survival (S_a_) is much lower than that in R, even across broad gradients in habitat quality, and R is often highly correlated with λ_h_, S_a_, and juvenile survival (S_j_; [5,54–56]). In a spotted owl (*Strix occidentalis*) population, for example, territory-specific S_a_ was nearly constant except at very low λ_h_ but R declined proportionally with λ_h_ across the full range of variation in λ_h_ [5]. Moreover, in a subset of patches where we monitored survival, R was correlated with S_j_ (*r* = 0.49, *n* = 32) and 2.5 ± 0.5 times lower and zero in 69% of cases where adult mortality occurred.

To estimate R, we located nests by observing owls, searching for sign, and with a pole-mounted video camera, which we also used to monitor nests, estimate nestling age, and time final visits immediately before fledging. We defined R as the number of young that survived to within one week of fledging in each occupied patch in each year, which is highly correlated with numbers that actually fledge (*r* = 0.93, *n* = 35). We considered R to be zero in occupied patches where no nest was found so long as all potential nests were checked, time between visits was not sufficient to complete nesting, or if behavior indicated failure to nest. We also considered R to be zero if nests were empty before young could have reached an age of 26 days, which is the earliest we observed successful fledging (most young fledge 28–30 days after hatching). If nests failed early and owls re-nested, we considered results of the last attempt.

### Ethics statement

All study sites were on private or communally-owned lands and were accessed with permission of landowners and community members. Research protocols for observational sampling were approved by the University of Arizona and University of Montana Institutional Animal Care and Use Committees. Ranch names can be obtained from the corresponding author.

### Hypotheses

We developed hypotheses to explain the effects of factors associated with each environmental component on R. To develop hypotheses and translate them into statistical models we used information on this and related systems, and considered three forms of most effects. Linear forms predicted effects changed at a constant rate, pseudo-threshold forms (ln + 1) predicted effects changed at a constant rate then approached an asymptote, and quadratic forms predicted maximal or minimal effects at intermediate values.

We developed 6 hypotheses to explain the effects of spatial factors on R based on the following themes: safe nest sites, environmental harshness, habitat amount, type, and configuration, energy, topographic complexity, and anthropogenic disturbance (S1 Appendix). Because safe sites are critical for nesting, we predicted R increased (e.g., linear or pseudo-threshold forms) with abundance of potential nests. Because environmental harshness can affect performance, we predicted R increased at cooler higher elevations, was greater in grasslands vs. more arid desert-scrub, or was greater at moderate elevations (e.g., quadratic form). Because foraging space and cover are critical for reproduction and prey, we predicted R increased with amounts of foraging, perching, and prey habitat in patches. Because we were unsure how best to represent habitat, we considered three definitions: woodland, woodland core area, and edge (Table 1). Because habitat configuration can affect performance independent of habitat amount [57], we predicted R declined as woodland habitat became more fragmented. Alternatively, because energy is a fundamental resource, we predicted R increased with net primary productivity (NPP) that we estimated by measuring normalized difference vegetation index (NDVI), which is highly correlated with NPP [58]. Finally, because the benefits of food may not be realized without safe nests, we predicted the effects of factors linked to food and foraging space (e.g., woodland amount) depended on nest-site abundance.

**Table 1:** Factors considered when modeling the effects of habitat resources on reproductive output of ferruginous pygmy-owls in northwest Mexico, 2001–2010.

Factor	Code	Definition	Units
Cavities	Cav	No. of saguaros with at least one suitable nesting cavity counted on a multiplicative scale (e.g., 1, 2, 4, 8, 16…)	no.
Vegetation Community	Comm	Dominant community type in patch (desert-scrub or semi-desert grassland).	categorical
Elevation	Elev	Mean elevation from digital elevation model	m
Woodland Habitat	Hab_f_	Mean fractional woody vegetation cover among all 30 × 30 m grid cells across patch	%
Hab_w_	Proportion of patch classified as woodland (e.g. 30 × 30 m grid cells with ≥20% fractional woody cover)	%
Core-Area Habitat	Core_hab_	Proportion of patch classified as woody vegetation minus 30 m edge width	%
Edge Habitat	Edge_total_	Length of edge between all 5 total land-cover classes	m
	Edge_hab_	Length of edge between woodland and other land-cover classes	m
Productivity	NDVI_mean_	Mean normalized-difference vegetation index measured every 16 days over 10 years	ratio × 1000
Substrate	Elev_cv_	Coefficient of variation in elevation among all 30 × 30 m grid cells	m
	Slope	Mean slope among all 30 × 30 m grid cells in patch	%
Disturbance	Disturb	Proportion of patch classified as agriculture, development, or road land-cover classes	%
Woodland Fragmentation	Frag_hab_	No. of patches of woodland per ha divided by Hab_f_	no./ha/%

Factors where quantified at the scale of individual territory patches (50 ha).

Topography and disturbance could influence R by affecting resources. In the Sonoran Desert, pygmy-owl’s main prey are various species of diurnal lizards that partition their use of the environment across a range of soil substrates [59]. Thus, we hypothesized patches with higher substrate diversity and hence more varied prey habitat affected R, and predicted R increased with increasing, or at moderate, topographic complexity. Because anthropogenic disturbance can degrade resources, we predicted R declined with increasing, or at moderate, disturbance.

In developing models to represent hypotheses, we first considered each potential definition of habitat, then the effects of topography and disturbance. Because an effect of habitat configuration was implicit when considering edge and core area, we considered fragmentation only when assessing the effect of woodland amount. We suspected safe nests and environmental harshness were important regardless of other factors, and thus considered them in all models.

We developed 5 hypotheses to explain the effects of temporal factors on R. Temperature (T) could have direct physiological effects on owls or indirect effects on resources and explain R in two general ways. If severe winters affect body condition or prey resources, we predicted R declined with lower average minimum winter T. If high T during nesting causes mortality of nestlings, limits activity of adults, or affects prey activity or abundance, we predicted R declined with increasing average maximum T during nesting. If precipitation (P) augments plant productivity and prey, as often occurs in arid regions [60], we predicted R increased with increasing P. If increasing NPP augments prey or other resources, we predicted R increased with increasing NDVI. If owls time breeding to coincide with favorable conditions, we predicted timing of peak NDVI explained R. We also considered models representing the combined effects of multiple hypotheses (S2 Appendix). Because the effect of weather factors could vary seasonally and interact, we considered average maximum T during incubation and brooding, annual, cool-season, and warm-season P and NPP (Table 2), and interactions between some factors. Finally, we hypothesized conspecifics had negative effects driven by intraspecific competition, and predicted R declined with presence and abundance of conspecifics.

**Table 2:** Factors considered when modeling the effects of weather and primary productivity on reproductive output of ferruginous pygmy-owls in northwest Mexico, 2001–2010.

Factor	Period	Code	Definition	Units
Temperature	Winter—recent	T_winter_	Mean daily minimum temperature Nov.—March	°C
	Incubation—current	T_incub_	Mean daily maximum temperature April	°C
	Brooding—current	T_brood_	Mean daily maximum temperature May and June	°C
Precipitation	Warm season—1 yr lag	P_ws_	Total precipitation June—Sept of prior year	cm
	Cool season—0.5 yr lag	P_cs_	Total precipitation Oct.—May, recent cool season	cm
	Annual—0–1 yr lag	P_yr_	Total precipitation recent cool season and prior warm season	cm
Primary Productivity	Warm season—1 yr lag	NDVI_ws_	Deviation from mean NDVI June—Sept of prior year	Proportion
	Cool season—0.5 yr lag	NDVI_cs_	Deviation from mean NDVI Oct—May, recent cool season	Proportion
	Annual—0–1 yr lag	NDVI_yr_	Deviation from mean NDVI recent cool season and prior warm season	Proportion
Timing of Primary Productivity	Warm season—1 yr lag	S_NDVIws_	Days since June 1 of maximum NDVI June—Sept of prior year	Day no.
	Cool season—0.5 yr lag	S_NDVIcs_	Days since Oct 1 of maximum NDVI Oct—May, recent cool season	Day no.

Primary productivity was quantified based on normalized difference vegetation index (NDVI) at the scale of individual territory patches (50 ha) whereas weather was quantified at the closest of five weather stations to each patch.

### Environmental measurements

We used remote-sensing and on-the-ground methods to quantify factors in spatial hypotheses (Table 1). Because saguaros were the only substrates used for nesting, we counted saguaros with potential to support cavities in each patch to estimate abundance of potential nests. To quantify elevation and slope, we used 30-m digital elevation models. To quantify NPP, we averaged patch-specific estimates of NDVI across all 10 years by compiling data (250-m resolution; see http://modis.gsfc.nasa.gov) every 16 days between 9 June 2000 and 25 May 2010 (*n* = 23 samples/yr) as area-weighted averages for each patch. NDVI ranged from 0.133–0.725 and cloud contamination was low (1.4%).

We used multiple methods to classify land cover into five classes (woodland, non-woodland, agriculture or other clearing, housing or development, or roadway corridor; S3 Appendix). We extracted spectral vegetation and soil data from 30-m resolution Landsat5 images and classified pixels with ≥20% woody vegetation cover as woodland, which given typical tree spacing distinguished open woodland and scrub from more closed-canopy woodland (S3 Appendix). To classify land cover representing disturbance, we used Google Earth imagery (GE) and digitized polygons around those features. We used those Landsat5- and GE-derived land cover data and program Fragstats [61] to estimate coverage of each land cover class in patches, woodland fragmentation, and amount of woodland core area and edge. To quantify woodland fragmentation independent of woodland amount, we scaled density of woodland patches by mean woody vegetation cover (Table 1). To quantity edge, we estimated edge length between all land cover classes and between woodland and other classes. To quantify amount of woodland core-area, we subtracted an edge width of 30 m from all woodland patches and computed the remaining area. Because landscape structure can affect performance, we also estimated the area of land cover classes that represented disturbance within 500 m of patches.

Data on negative heterospecific interactions are useful for evaluating the functional roles of important resources. Thus, we considered interactions with two larger cavity nesters (western screech-owl, *Megascops kennicottii*; American kestrel, *Falco sparverius*) by noting evidence of mortalities caused by these species or instances where they appropriated nests used by pygmy-owls. In the case of mortalities, owls were found decapitated but not consumed near nests.

We used satellite and weather-station data to quantify factors associated with temporal hypotheses (Table 2). For weather, we used data on monthly P and monthly minimum and maximum T from the closest of five weather stations to each patch (see http://www.wrcc.dri.edu; Fig. 1). To quantify temporal variation in NPP independent of spatial variation, we calculated proportional deviations from mean NDVI during each season or year where NDVI deviation = (mean NDVI for the season or year—mean NDVI for the period in all years)/mean NDVI for the period in all years. For timing of peak NPP, we calculated the number of days between maximum NDVI in the warm and cool seasons and the start of those seasons (Table 2).

To describe presence and abundance of conspecifics at two large scales, we used survey data to calculate the proportion of patches occupied each year in the study area and in each of 11 watershed regions (Fig. 1; S4 Appendix). At a local scale, we estimated presence, number, and density of territorial pygmy-owls around each focal patch. To estimate local density (territories/km^2^), we considered the number (*n*) and average distance in m ($\overset{-}{D}$) to nearest-neighbor nests as

$$\frac{1\;x\;10^{6}\;m^{2}}{\left(\left({\overset{-}{D}}^{2}\right)\times \left(\frac{1}{n}\right)\right)}$$ (1)

Thus, estimates of local density (*sensu* [62]) were based on the number and proximity of conspecific neighbors, which is simple to measure in this system because most nests have 0–2 nearest neighbors due to the linear arrangement of woodland along drainages.

### Analyses

#### Modeling approach

As a general approach, we compared models representing hypotheses associated with each environmental component, and then assessed the combined effects of multiple components. To evaluate support among models, we used an information-theoretic approach and Akaike’s information criterion adjusted for sample size (AIC_c_) and AIC_c_ weights (*w*_*i*_) to compare models [63]. Models within approximately 2 ΔAIC_c_ units were considered competitive except when they included uninformative parameters [63].

We used linear mixed-effects (LME) models to estimate fixed and random effects, process variance (σ^2^_process_), and residual variance (σ^2^_ε_) [64]. Before modeling fixed effects, we used an over-fitted model, restricted maximum likelihood (REML), and AIC_c_ to select optimal forms of the random effects and variance-covariance matrices. As random effects, we considered models with territory patch fit as a random intercept, which ensured standard errors for fixed effects were based on the number of patches not observations, and with crossed random effects for patch and year. To assess potential heterogeneity in σ^2^_ε_, we considered models with one variance, variances for each year, and variance covariates that could affect the range of patch qualities used across time. No spatial or temporal correlation structures were used because autocorrelation functions indicated temporal autocorrelation was low and variograms indicated no spatial autocorrelation.

We followed three steps when developing models with different fixed effects. First, we selected the best model to represent each hypothesis by comparing models in each suite of related models with similar factors (e.g., seasonal vs. annual P), interactions, and effect forms. Second, we used AIC_c_ to rank models representing each hypothesis. Finally, we refined the best models by assessing the effects of including or excluding some factors and interactions. When refining models, we considered correlations between factors, which were low in all cases (*r* ≤ 0.41).

We fit models with the nlme library in R and used maximum likelihood (ML) methods to estimate fixed effects. We used a Gaussian-based approach because it is more robust than generalized linear models when data do not conform to Poisson or negative binomial distributions, which fit our data poorly due to few broods of 1–2 young [65]. Regardless, zero-inflation was low (22%), diagnostic tools indicated models met all assumptions, and all predictions were positive.

#### Relative and combined effects of components

We used two approaches to assess the relative and combined effects of each component. In a model selection framework, we combined the best models for the effects of habitat, weather, and conspecifics into all possible combinations of additive models, which produced seven models. We also considered models with all possible combinations of interactions among components, which produced another seven models. To represent models with interactions, we used AIC_c_ to select the best models among all possible combinations of 2-way interactions between factors.

To assess the relative contribution of each component, we used components of variance analyses [66] to decompose process variance into spatial and temporal components and to estimate the proportion of variance in R explained by each component. Spatial and temporal process variation in R can be decomposed as σ^2^_process_ = σ^2^_spatial_ + σ^2^_temporal_. To estimate σ^2^_spatial_, we used an intercepts-only model with a random intercept for territory patch, the best variance-covariance structure, and REML. To estimate σ^2^_temporal_, we used the same approach but fit year as a random intercept. Magnitudes of spatial vs. temporal process variance were then expressed as ratios, proportions (e.g., σ^2^_spatial_/σ^2^_process_), and coefficients of process variation (CV)

$$\frac{\sqrt{\sigma_{\text{process}}^{2}}}{\overset{-}{R}}$$ (2)

where $\overset{-}{R}\;$ is mean R among years or patches and σ^2^_process_ is spatial or temporal process variance.

To estimate amounts of variation explained by important fixed effects associated with each component, we partitioned process variance as σ^2^_process_ = σ^2^_model_ + σ^2^_residual_ where σ^2^_process_ is either total spatial or temporal variation in R, σ^2^_model_ is the amount of that variation explained by the best model for either habitat or weather factors (e.g., σ^2^_habitat_ and σ^2^_weather_), and σ^2^_residual_ is unexplained variance. Total process variation explained by habitat or weather was then estimated as σ^2^_model_ = σ^2^_process−_σ^2^_residual_. In the LME approach used here, we estimated σ^2^_process_ using an intercepts-only model, REML, and the best variance-covariance structures. To estimate σ^2^_residual_, we fit our best model for spatial and temporal factors using REML, which provides unbiased estimates of variance not explained by fixed effects [66]. Because conspecifics affect R across both space and time, we further decomposed σ^2^_process_ to assess the proportion of additional variation explained by conspecifics by combining our best model for conspecifics with that for habitat and weather, and repeating procedures described above.

Total variation in R explained by the environment was expressed as σ^2^_total_ = σ^2^_habitat_ + σ^2^_weather_ + σ^2^_conspecifics_ = σ^2^_model_ + σ^2^_residual_ where σ^2^_habitat_, σ^2^_weather_, and σ^2^_conspecifics_ are estimates of variation due to habitat, weather, and conspecifics, σ^2^_model_ is the amount of that variation explained by a model with those effects, and σ^2^_residual_ is unexplained variation. Because conspecifics affect habitat quality in both space and time, we estimated σ^2^_conspecifics_ assuming σ^2^_model_ = σ^2^_process—_σ^2^_residual_ and computed σ^2^_model_ by summing estimates from both temporal and spatial models that included the effect of conspecifics. To estimate relative contributions of each component, we expressed the proportion of σ^2^_model_ attributable to each component as σ^2^_*x*_/σ^2^_model_, where *x* is habitat, weather, or conspecifics. Because the effect of conspecifics may depend on the spatial arrangement of habitat, we preformed analyses for the entire population and for only those patches with conspecific neighbors.

## Results

We identified 107 territory patches over 10 years of which 56% were in desert-scrub (vs. grassland) and 89% were monitored for ≥7 years. We obtained an average of 4.4 ± 0.2 (± SE) estimates of R per patch (*n* = 468), ≥3 estimates in 73% of patches, and only single estimates in 14% of patches that were rarely occupied. We obtained an average of 46.8 ± 4.3 estimates/year, and ≥43 estimates/year except in 2001 (*n* = 32) and 2003 (*n* = 18).

A model with territory patch fit as a random intercept and a single residual variance were the best approximating structures vs. models with crossed random effects for patch and year (ΔAIC_c_ = 2.20), residual variances for each year (ΔAIC_c_ = 7.41), or variance covariates (ΔAIC_c_ ≥ 9.25). Those structures were optimal in all models.

### Spatial factors

R averaged 2.65 ± 0.11 young per occupied patch and varied markedly across space (*F*_106, 361_ = 1.32, *P* = 0.032, ANOVA). Spatial process variance (σ^2^_spatial_; 0.216) and a coefficient of spatial process variation (0.176) were relatively high. When the effects of important habitat factors were considered, patch-specific predictions of R varied >4 fold (0.91–3.97) across space.

The best model of the effects of spatial factors was {lnCav + Comm + Hab_f_ + lnCav*Hab_f_ + Frag_hab_} (model 3 in Table 3). This model represented the hypotheses that abundance of potential nest sites, environmental harshness, and amount and configuration of woodland habitat explained R by affecting food, foraging space, and vulnerability to physiological stress and heterospecific enemies. This model included a positive effect of semi-desert grassland, a negative effect of woodland fragmentation, and an interaction between amount of woodland habitat and abundance of potential nest sites. Two others models received some support. One model (model 4) included the same factors as the best model and an interaction between slope and abundance of potential nest sites. Another model (model 11) hypothesized overall NPP explained R and included a positive effect of semi-desert grassland and an interaction between mean NDVI and abundance of potential nest sites.

**Table 3:** Rankings and estimated slope parameters for hypothesized models that explained the effects of habitat factors on reproductive output of ferruginous pygmy-owls in northwest Mexico, 2001–2010.

Model		Parameter estimates (β ± SE)	K	LL	ΔAICc	w_*i*_
3)	lnCav + Comm + Hab_f_ + lnCav*Hab_f_ + Frag_hab_	0.15 ± 0.20, 0.46 ± 0.16, -0.068 ± 0.034, 0.023 ± 0.010, -0.18 ± 0.085	8	-896.87	0.00	0.237
4)	lnCav + Comm + Hab_f_ + lnCav*Hab_f_ + Slope + lnCav*Slope + Frag_hab_	-0.17 ± 0.26, 0.38 ± 0.18, -0.069 ± 0.034, 0.023 ± 0.010, -0.66 ± 0.44, 0.27 ± 0.15, -0.19 ± 0.086	10	-894.79	0.01	0.235
11)	lnCav + Comm + NDVI_mean_ + lnCav*NDVI_mean_	-0.55 ± 0.53, 0.50 ± 0.16, -1.0 ± 0.57, 0.41 ± 0.19	7	-898.63	1.47	0.114
B)	lnCav + Comm	0.57 ± 0.085, 0.54 ± 0.16,	5	-901.39	2.86	0.057
2)	lnCav + Comm + Hab_f_ + lnCav*Hab_f_ + Slope + lnCav*Slope + Disturb	-0.060 ± 0.26, 0.51 ± 0.18, -0.050 ± 0.033, 0.018 ± 0.010, -0.77 ± 0.44, 0.27 ± 0.15, 0.11 ± 0.078	10	-896.23	2.90	0.056
1)	lnCav + Comm + Hab_f_ + lnCav*Hab_f_ + Slope + lnCav*Slope	-0.063 ± 0.26, 0.45 ± 0.18, -0.045 ± 0.033, 0.018 ± 0.010, -0.71 ± 0.44, 0.27 ± 0.15	9	-897.30	2.95	0.054
8)	lnCav + Comm + Edge_tot_	0.60 ± 0.087, 0.54 ± 0.16, 0.063 ± 0.047	6	-900.51	3.16	0.049
7)	lnCav + Comm + Core_hab_ + lnCav*Core_hab_ + Slope + lnCav*Slope + Disturb	0.039 ± 0.23, 0.51 ± 0.18, -0.34 ± 0.24, 0.13 ± 0.077, -0.81 ± 0.44, 0.28 ± 0.15, 0.11 ± 0.077	10	-896.43	3.30	0.045
5)	lnCav + Comm + Core_hab_ + lnCav*Core_hab_	0.38 ± 0.14, 0.49 ± 0.16, -0.31 ± 0.24, 0.13 ± 0.077,	7	-899.57	3.34	0.045
4)	lnCav + Comm + Core_hab_ + lnCav*Core_hab_ + Slope + lnCav*Slope	0.030 ± 0.23, 0.44 ± 0.17, -0.31 ± 0.240.13 ± 0.077, -0.75 ± 0.44, 0.29 ± 0.15	9	-897.55	3.45	0.042
9)	lnCav + Comm + Edge_tot_ + Slope + lnCav*Slope	0.25 ± 0.21, 0.51 ± 0.17, 0.042 ± 0.049, -0.75 ± 0.44, 0.27 ± 0.15	8	-898.83	3.92	0.033
10)	lnCav + Comm + Edge_tot_ + Slope + lnCav*Slope + Disturb	0.23 ± 0.21, 0.58 ± 0.18, 0.020 ± 0.052, -0.83 ± 0.45, 0.28 ± 0.15, 0.11 ± 0.079	9	-897.81	3.96	0.033
	β_0_ + b_0i_		3	-923.08	42.17	0.000

Definitions of factors are in Table 1 and descriptions of hypotheses are in S1 Appendix. The intercepts-only model (β_0_+ b_0*i*_) is included for comparison

Evidence for an effect of woodland habitat was stronger than that for edge or woodland core-area, and likelihoods of models that included those factors were ≥4.8 times lower (Table 3). Although R increased somewhat with increasing edge, there was little evidence of an effect of edge when considered in the best model (Table 4). The effect of woodland habitat was better represented by mean woody vegetation cover than by the proportion of patches classified as woodland (∆AIC_c_ = 1.46).

**Table 4:** Rankings of best approximating models of the effects of temporal and spatial factors on reproductive output of ferruginous pygmy-owls in northwest Mexico, 2001–2010 compared to other models where some effects were included, excluded, or changed.

Change in effects	Model	ΔAICc	*w*_*i*_
Temporal factors			
Best approximating model	lnT_brood_ + lnP_yr_ + lnT_brood_*lnP_yr_ + NDVI_yr_^2^	0.00	0.436
Inclusion of quadratic time effect	Year^2^ + lnPyr + lnTbrood*lnPyr + NDVI_yr_^2^	1.57	0.199
Inclusion of linear time effect	Year + lnP_yr_ + lnTb_rood_*lnP_y_r + NDVI_yr_^2^	1.58	0.198
Exclusion of P*T interaction	lnT_brood_ + lnP_yr_ + NDVI_yr_^2^	3.33	0.083
Exclusion of NDVI effect	lnT_brood_ + lnP_yr_ + lnT_brood_*lnP_yr_	3.80	0.065
Intercepts only model	β_0_ + b_0*i*_	7.76	0.009
Quadratic time effect only	Year^2^	8.92	0.005
Linear time effect only	Year	9.13	0.005
Spatial factors			
Best approximating model	lnCav + Comm + Hab_f_ + lnCav*Hab_f_ + Frag_hab_	0.00	0.266
Inclusion of Disturb effect	lnCav + Comm + Hab_f_ + lnCav*Hab_f_ + Frag_hab_ + Disturb	0.31	0.227
Exclusion of Hab effect	lnCav + Comm + Frag_hab_	0.93	0.167
Inclusion of Edge effect	lnCav + Comm + Hab_f_ + lnCav*Hab_f_ + Frag_hab_ + Edge_total_	1.02	0.159
Inclusion of quadratic Elev effect	lnCav + Comm + Hab_f_ + lnCav*Hab_f_ + Frag_hab_ + Elev^2^	1.90	0.103
Exclusion of Hab*Cav interaction	lnCav + Comm + Hab_f_ + Frag_hab_	2.88	0.063
Exclusion of Comm effect	lnCav + Hab_f_ + lnCav*Hab_f_ + Frag_hab_	5.72	0.015
Exclusion of lnCav effect	Comm + Hab_f_ + Frag_hab_	38.17	0.000

**Fig 2:**
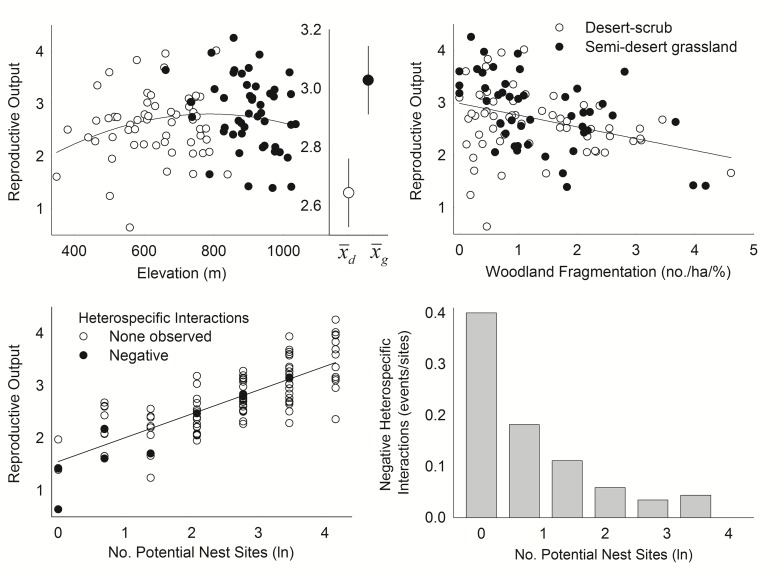
Interactive effects of abundance of potential nest sites and other habitat factors on reproductive output of ferruginous pygmy-owls in northwest Mexico, 2001–2010. Estimates of reproductive output are based on the top-ranked models that include each of the habitat factors represented as summarized in Table 3.

**Fig 3:**
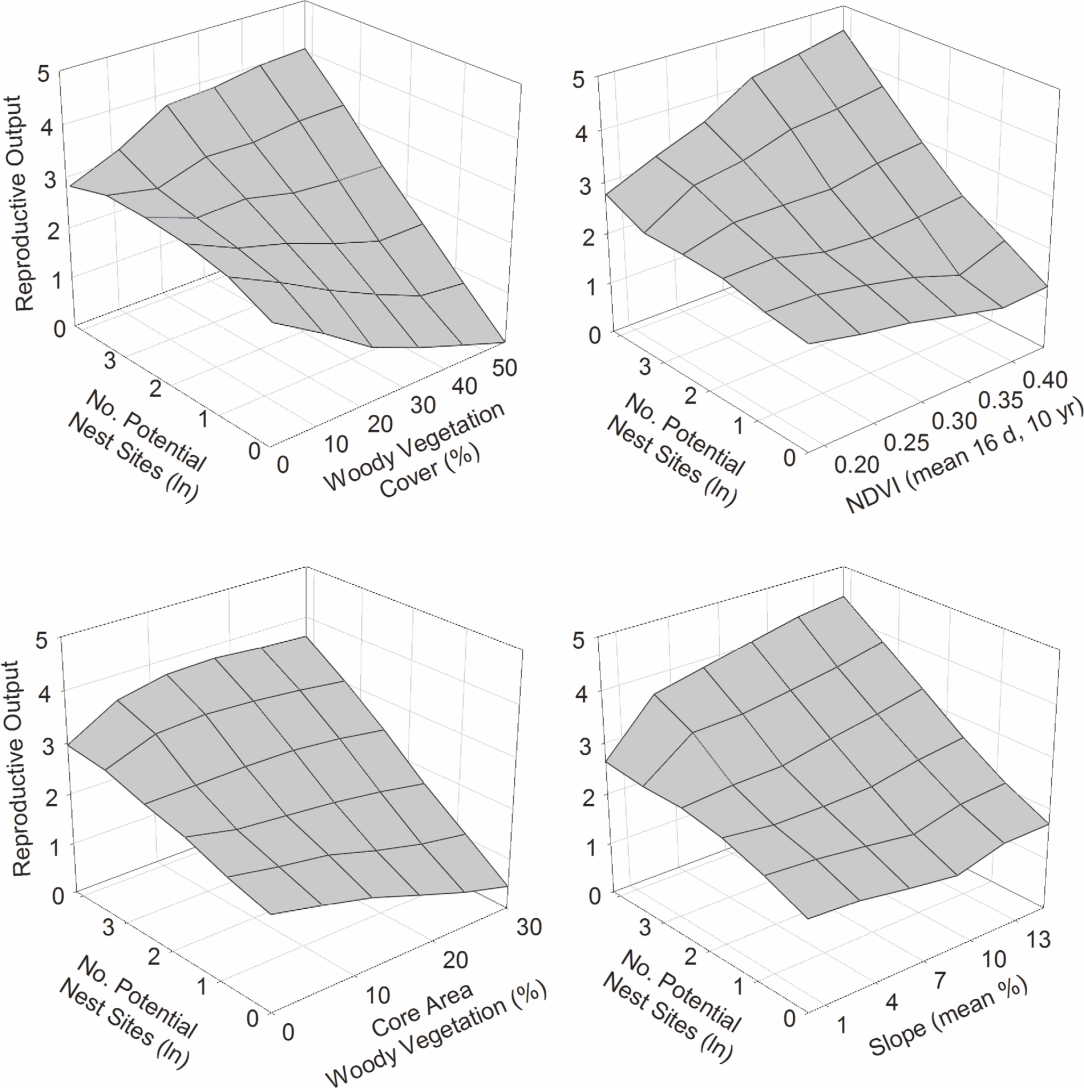
Effect of habitat factors on reproductive output of ferruginous pygmy-owls in northwest Mexico, 2001–2010. Lower right figure shows the number of negative heterospecific interactions observed divided by the total number of territory patches in each group across a gradient of increasing abundance of potential nest sites. Filled circles in upper figures are patches in semi-desert grasslands whereas those in the lower figure are patches where we observed negative heterospecific interactions. Estimates of reproductive output are based on model 3 in Table 3. Inset in upper left figure shows means (± SE) in each vegetation community.

The effects of factors related to food and foraging space depended largely on abundance of potential nest sites (Fig. 2), which had a large effect. R increased markedly with increasing nest-site abundance (2.2 ± 0.4/young increase across the full range of variation; Fig. 3), but its effect was best represented by an interaction with amount of woodland habitat (Table 4). R increased markedly with nest-site abundance only in patches with moderate to high amounts of woodland but much less otherwise. Moreover, this same general pattern applied to other factors linked to food and foraging space; once nest-site abundance reached moderate levels, R increased with increasing amount of woodland habitat, NDVI, and slope, but with weaker effects of woodland core area (Fig. 2). Where nest-site abundance was low, however, amount of woodland habitat had negative effects on R.

**Fig 4:**
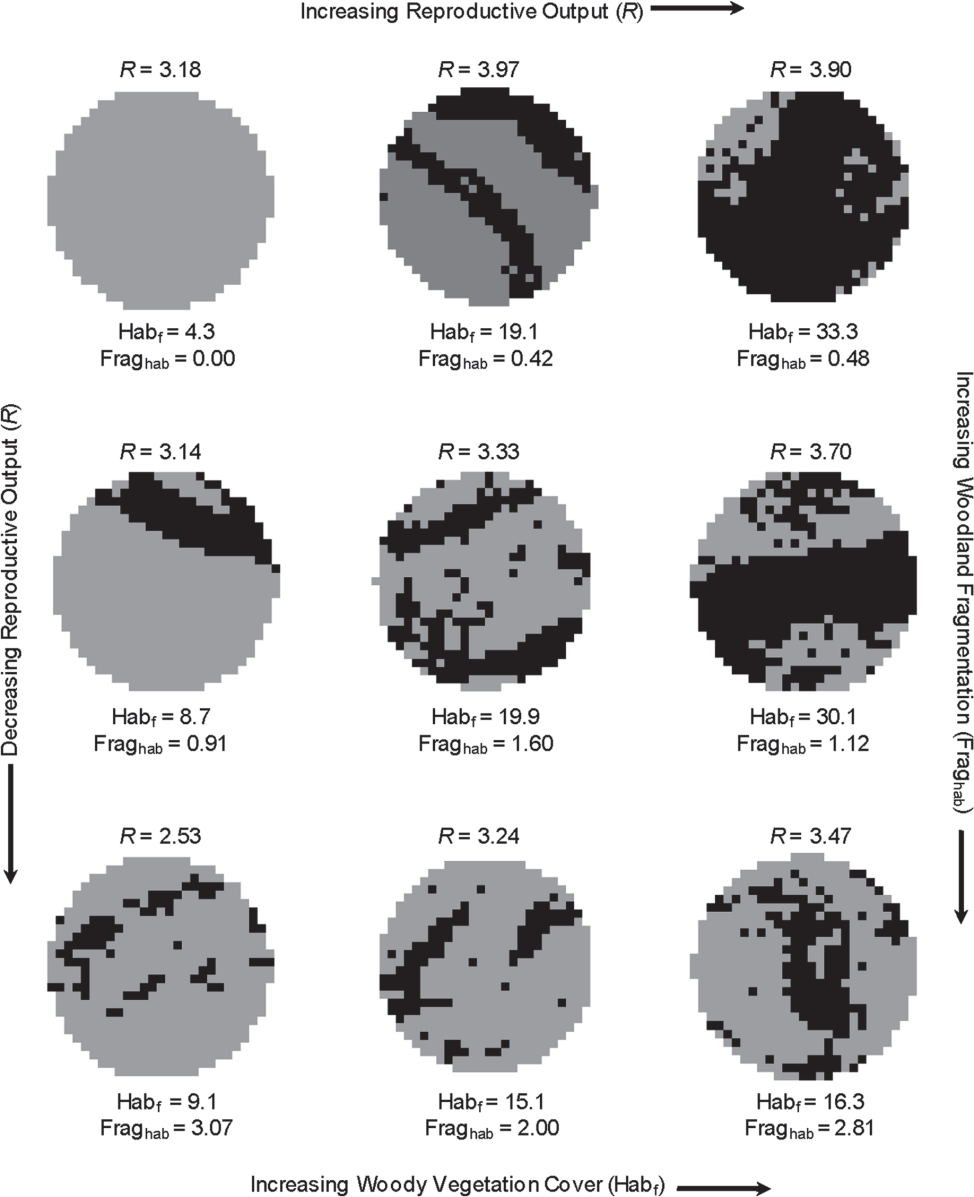
Effects of habitat frgamentation (Frag_hab_) and quantity of woodland vegetation cover (Hab_f_) on reproductive output (*R*) of ferruginous pygmy-owls in northwest Mexico, 2001–2010. The 9 territory patches shown all have high abundance of potential nest sites and were selected to illustrate effects. Black pixels (30-m) had ≥20% woody vegetation cover and were classified as woodland and gray pixels had <20% woody vegetation cover. Estimates of *R* are based on model 3 in Table 3.

Woodland fragmentation but not anthropogenic disturbance affected R (Table 4). On average, R decreased with increasing woodland fragmentation (Figs. 3–4), which was not highly correlated with woodland amount (*r* = 0.41).

Macrohabitats (e.g., vegetation communities) had important effects on R, which averaged ≥0.38 ± 0.16 higher in grasslands than in desert-scrub, after adjusting for other factors (Table 3; Fig. 2). Although R was higher on average at moderate elevations (Fig. 3), macrohabitat effects provided a better explanation of the data than the continuous, nonlinear effect of elevation or both factors combined (Table 4). Macrohabitat effects were likely not driven by associations with other important factors because those factors either did not vary between macrohabitats (*p* ≥ 0.77, *t*-tests for NDVI_mean_ and Frag_hab_) or were greater in desert-scrub (*p* ≤ 0.051, lnCav and Hab_f_). Although magnitudes of slope parameters for important effects were often similar in both macrohabitats when assessed independently, R declined with increasing woodland fragmentation at a much greater rate in grasslands (*β* ± SE = -0.35 ± 0.088) than in desert-scrub (-0.10 ± 0.085; least squares regression). Macrohabitat effects were likely linked to environmental harshness because decadal differences in annual precipitation (P) and brooding-season temperature (T) averaged 44.8 ± 6.0% higher and 3.1 ± 1.0% lower in grassland, respectively.

We found evidence of negative heterospecific interactions in 7.5% of patches and 92% were with western screech-owl. Prevalence of such interactions decreased markedly as abundance of potential nest sites increased (Fig. 3). Where nest substrates were rare, woodland cover averaged 51.7 ± 26.3% higher in patches where we observed negative heterospecific interactions.

### Temporal factors

**Fig 5:**
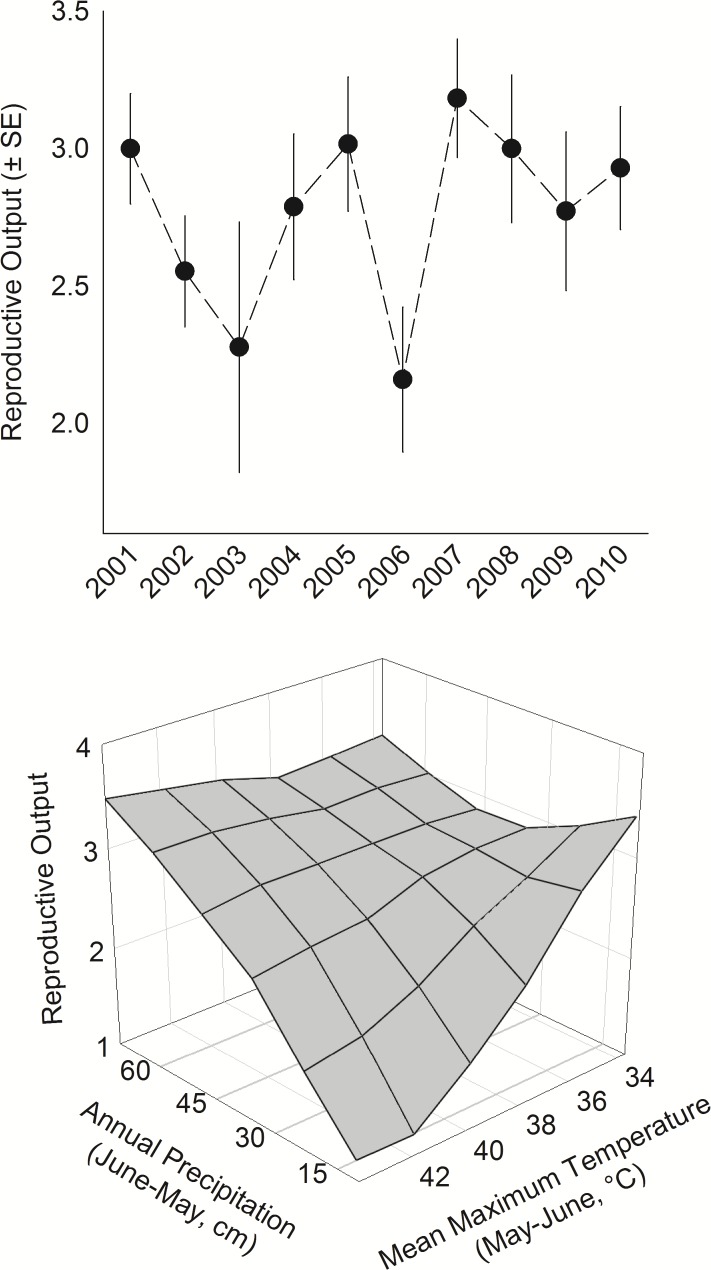
Temporal variation and effects of weather on reproductive output of ferruginous pygmy-owls in northwest Mexico, 2001–2010. Temperature and precipitation were measured at regional scales and estimates are from model 8 in Table 5.

Annual estimates of R averaged 2.77 ± 0.11 young per occupied patch and varied somewhat across time (range = 2.16–3.18, *F*_9, 458_ = 1.59, *P* = 0.116; Fig. 5). Temporal process variance (σ^2^_temporal_; 0.0380) and a coefficient of temporal process variation (0.0703) were relatively low.

**Table 5:** Rankings and estimated slope parameters for hypothesized models that explained the effects of temporal factors on reproductive output of ferruginous pygmy-owls in northwest Mexico, 2001–2010.

Model		Parameter estimates (β ± SE)	K	LL	ΔAICc	*w*_*i*_
8)	lnT_brood_ + lnP_yr_ + lnT_brood_*lnP_yr_ + NDVI_yr_^2^	-33.6 ± 14.0, -31.0 ± 13.6, 8.7 ± 3.7, 20.3 ± 9.2	7	-915.10	0.00	0.318
11)	lnT_brood_ + lnP_yr_ + lnT_brood_*lnP_yr_ + NDVI_yr_^2^ + S_NDVIws_^2^	-33.5 ± 14.0, -31.0 ± 13.6, 8.6 ± 3.7, 20.6 ± 9.2, 0.30 ± 0.30	8	-914.43	0.72	0.222
12)	lnP_yr_ + NDVI_yr_^2^	0.44 ± 0.19, 18.6 ± 9.2	5	-918.26	2.19	0.106
14)	lnP_yr_ + NDVI_yr_^2^ + S_NDVIws_^2^	0.42 ± 0.19, 19.0 ± 9.2, 0.30 ± 0.30	6	-917.53	2.80	0.078
6)	lnT_brood_ + lnP_yr_ + lnT_brood_*lnP_yr_	-31.6 ± 14.0, -29.2 ± 13.6, 8.2 ± 3.7	6	-917.54	2.81	0.078
9)	lnT_brood_ + lnP_yr_ + lnT_brood_*lnP_yr_ + S_NDVIws_^2^	-31.5 ± 14.0, -29.2 ± 13.6, 8.1 ± 3.7, 0.30 ± 0.30	7	-916.95	3.69	0.050
3)	lnP_yr_	0.44 ± 0.19	4	-920.30	4.23	0.038
13)	lnP_yr_ + S_NDVIws_^2^	0.43 ± 0.19, 0.29 ± 0.26	5	-919.67	5.01	0.026
4)	NDVI_yr_^2^	19.9 ± 9.3	4	-920.99	5.62	0.019
15)	NDVI_yr_^2^ + S_NDVIws_^2^	19.4 ± 9.3, 0.34 ± 0.25	5	-920.11	5.90	0.017
7)	lnT_brood_ + NDVI_yr_^2^	-1.8 ± 1.4, 19.5 ± 9.3	5	-920.21	6.10	0.015
10)	lnT_brood_ + NDVIyr^2^ + S_NDVIws_^2^	-1.7 ± 1.4, 19.9 ± 9.3, 0.32 ± 0.25	6	-919.40	6.54	0.012
	β_0_ + b_0*i*_		3	-923.08	7.76	0.007
5)	S_NDVIws_^2^	0.32 ± 0.26	4	-922.30	8.24	0.005
2)	lnT_brood_	-1.7 ± 1.4	4	-922.41	8.46	0.005
1)	T_winter_	-0.0057 ± 0.029	4	-923.06	9.76	0.002

Definitions of factors are in Table 2 and descriptions of hypotheses are in S2 Appendix. The intercepts-only model (β_0_+ b_0*i*_) is included for comparison. Slope estimates and SE for S_NDVIws_ were multiplied by 100.

The best model of the effects of temporal factors was {lnT_brood_ + lnP_yr_ + lnT_brood_*lnP_yr_ + NDVI_yr_^2^} (model 8 in Table 5). This model represented the hypotheses that high T during nesting, and both annual P and NPP before nesting, explained R through direct or indirect pathways. Likelihood of a reduced model without the effect of T, and thus an interaction between T and P, was 3 times lower (Table 5). There was no evidence of an effect of timing of peak NPP, linear or non-linear temporal trends in R, or for the intercepts-only model (Table 4 and 5).

Definitions of factors are in Table 2 and descriptions of hypotheses are in S2 Appendix. The best model included a quadratic effect of annual NDVI deviation and an interaction between annual P and brooding-season T. On average, R was low or moderate during periods of low NDVI but increased rapidly at high NDVI. Although when considered independently, annual P had marked effects on R (0.30 ± 0.13 young increase with each doubling of P), the effect of P was best described by its interaction with brooding-season T. R increased markedly with increasing P during periods of high to moderate T, P had little effect during periods of low T, and importantly, R decreased to very low levels during periods of low P and high T (Fig. 5). R was very low in 2002 and especially in 2006 when annual P averaged only 28.7 ± 5.8 and 19.0 ± 3.3 cm, respectively, which was 19–46% lower than the decadal average. In 2006, which was among the hottest years on record in the region, brooding-season T averaged 38.9 ± 1.1°C or 4.7% higher than the decadal average. Annual P and brooding-season T were uncorrelated (*r* = -0.10, *P* = 0.49).

Seasonal periods selected to describe the effects of T and P were strongly supported by the data. Substituting cool-season P for annual P in the best model increased AIC_c_ by 8.41, but there was some support for an effect of warm-season P (AIC_c_ = 1.96). Substituting incubation-season T for brooding-season T increased AIC_c_ by 4.65.

### Conspecifics

Conspecifics occupied areas around 73.8% of focal patches in at least one year, but nested within 1.5 km of focal patches during only 43.6% of observations. Both the number and density of conspecifics around focal patches varied across space (*F*_106, 361_≥ 5.14, *P*< 0.001), with densities ranging from 0.0–5.5 territories/km^2^ ($\overset{-}{x\;}$ = 0.68 ± 0.04) and distances between nearest-neighbor nests ranging from 425–2,619 m ($\overset{-}{x\;}$ = 1,251 ± 33, *n* = 287). Conspecific density also varied across time (*F*_9, 458_ = 2.36, *P*< 0.001), with annual means that varied >2.5 fold (0.38–1.01).

**Fig 6:**
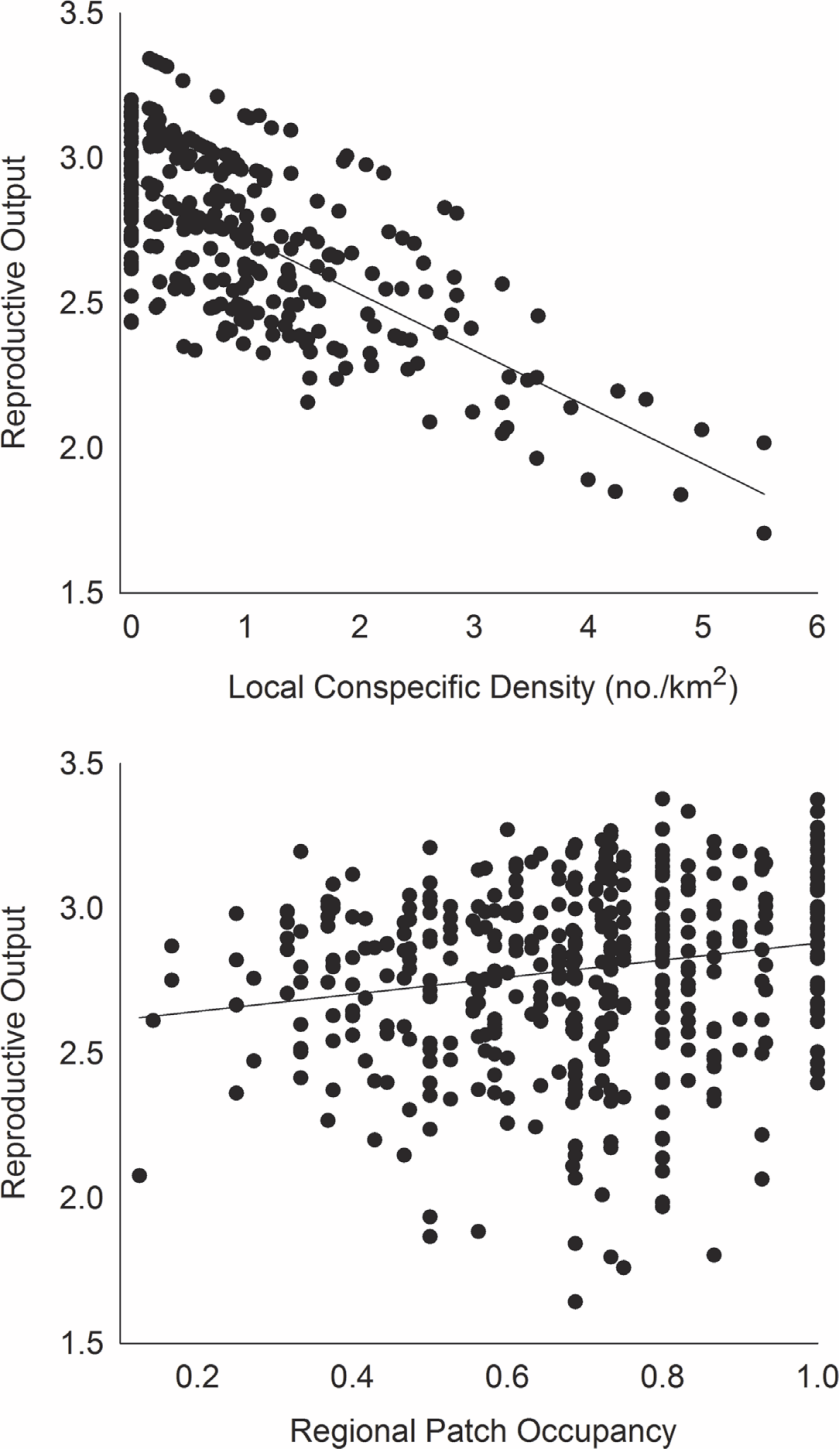
Effects of conspecifics on reproductive output of ferruginous pygmy-owls in northwest Mexico, 2001–2010. Conspecific density was measured around each focal patch each year and regional occupancy was measured as the proportion of patches occupied in each of 11 watershed regions in each year. Estimates of the effect of local conspecific density are based on model {Density} and estimates of the effect of regional occupancy are adjusted for the effects of local density from model {Density + Occ_region_} in S4 Appendix.

The effect of conspecifics was best described by factors measured at a local scale, and more specifically, by local conspecific density (S4 Appendix). R declined by 0.18 ± 0.084 young with each 1-territory/km^2^ increase in local density, and although R also declined with presence and number of conspecific neighbors, density had a much stronger effect (Fig. 6, S4 Appendix). Interestingly, after considering the effect of local density, R increased by 0.070 ± 0.042 young with each 10% increase in regional occupancy (Fig. 6).

### Relative contribution of components

Spatial process variance in R was 5.7 times greater than temporal process variance and 85.0% of process variance was attributable to space. Habitat factors in the best model explained >99.9% of spatial process variance but only 3.7% of residual variance, and σ^2^_habitat_ equaled 0.321. Weather factors in the best model explained >99.9% of temporal process variance but only 1.3% of residual variance, and σ^2^_weather_ equaled 0.0779. Thus, when σ^2^_conspecifics_ (0.0597) was included, σ^2^_model_ equaled 0.459 and the relative contribution of habitat, weather, and conspecifics in explaining R was 0.70, 0.17, and 0.13, respectively.

When the relative effects of each component were evaluated further, habitat effects were consistently strong but the effects of conspecifics and temporal factors varied. When considered with habitat factors, conspecifics explained little additional spatial variance (4.0 vs. 3.7%) and σ^2^_habitat_ increased by only 2.2%. When considered with temporal factors, however, conspecifics explained much more temporal variance (3.6 vs. 1.3%) and σ^2^_weather_ increased 87.4%. When only patches with conspecifics neighbors were considered, σ^2^_model_ increased to 0.555 and more variation was explained by habitat (0.82) than by weather (0.09) or conspecifics (0.09).

When assessed in a model-selection framework, evidence of habitat effects was much greater than that for weather or conspecifics, but all components were important in explaining R (Table 6). Likelihood of a model that included all factors in the best models for each component was 125 times higher than that for the habitat-only model (Table 6). Although relative support for an effect of conspecifics was lowest overall, likelihood of a model that included conspecifics was 4.8 times higher than a model that included only habitat and weather effects.

**Table 6:** Rankings of models that described the individual, additive, and interactive effects of spatial and temporal factors and conspecifics on reproductive output of ferruginous pygmy-owls in northwest Mexico, 2001–2010.

Hypothesis	K	LL	ΔAICc	*w*_*i*_
Habitat × Weather + Density	16	-882.99	0.00	0.264
Habitat + Weather × Density	14	-885.20	0.15	0.245
Habitat × Weather × Density	21	-877.83	0.55	0.201
Habitat + Weather + Density	13	-886.80	1.22	0.144
Habitat × Density + Weather	16	-884.41	2.84	0.064
Habitat × Weather	15	-885.76	3.40	0.048
Habitat + Weather	12	-889.43	4.36	0.030
Habitat × Density	12	-892.39	10.29	0.002
Habitat + Density	9	-895.75	10.72	0.001
Habitat only	8	-896.87	10.87	0.001
Weather × Density	9	-908.67	36.56	0.001
Weather + Density	8	-910.41	37.95	0.001
Weather only	7	-915.10	45.27	0.001
Density only	4	-920.79	50.49	0.001

Factors included in models for each component are those in the best approximating models. Models and parameter estimates are in S5 Appendix.

**Fig 7:**
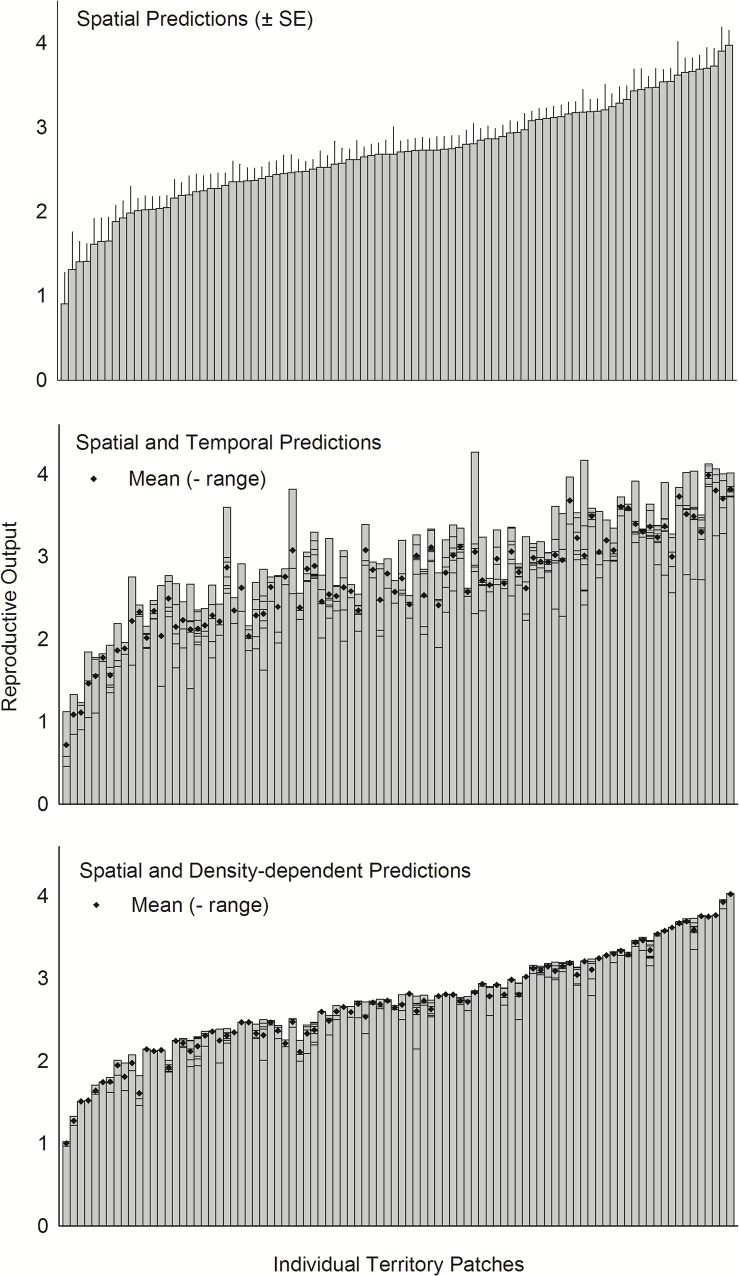
Estimated reproductive output within individual territory patches occupied by ferruginous pygmy-owls in northwest Mexico, 2001–2010. Patches are sorted in ascending order basis on the estimated habitat effects and only patches with ≥2 observations (*n* = 92) are shown. Upper figure shows predictions based on estimated habitat effects only (model 3, Table 3), and the middle and lower figures show estimates based on habitat and temporal factors, and habitat and conspecific density, respectively. In lower figures, diamonds are average reproductive output and horozontal lines across bars illustrate the range of estimates among years.

When the effects of habitat factors were considered, spatial variation in patch-specific predictions of R was high, and R initially increased very rapidly and more gradually thereafter (Fig. 7). When the additive effects of habitat and weather were considered, those same general patterns remained but weather effects re-ordered the relative quality of patches somewhat (Fig. 7). In some years, favorable weather amplified R by ≤56%, harsh weather depressed R by ≤49%, and the absolute value of weather effects averaged 10.5 ± 0.4%. In contrast, when the additive effects of habitat and conspecifics were considered, patch-specific predictions of R varied much less, changes in density amplified R by ≤13% or depressed it by ≤27%, and the absolute value of conspecific effects averaged only 3.3 ± 0.2%.

### Interactions among components

**Fig 8:**
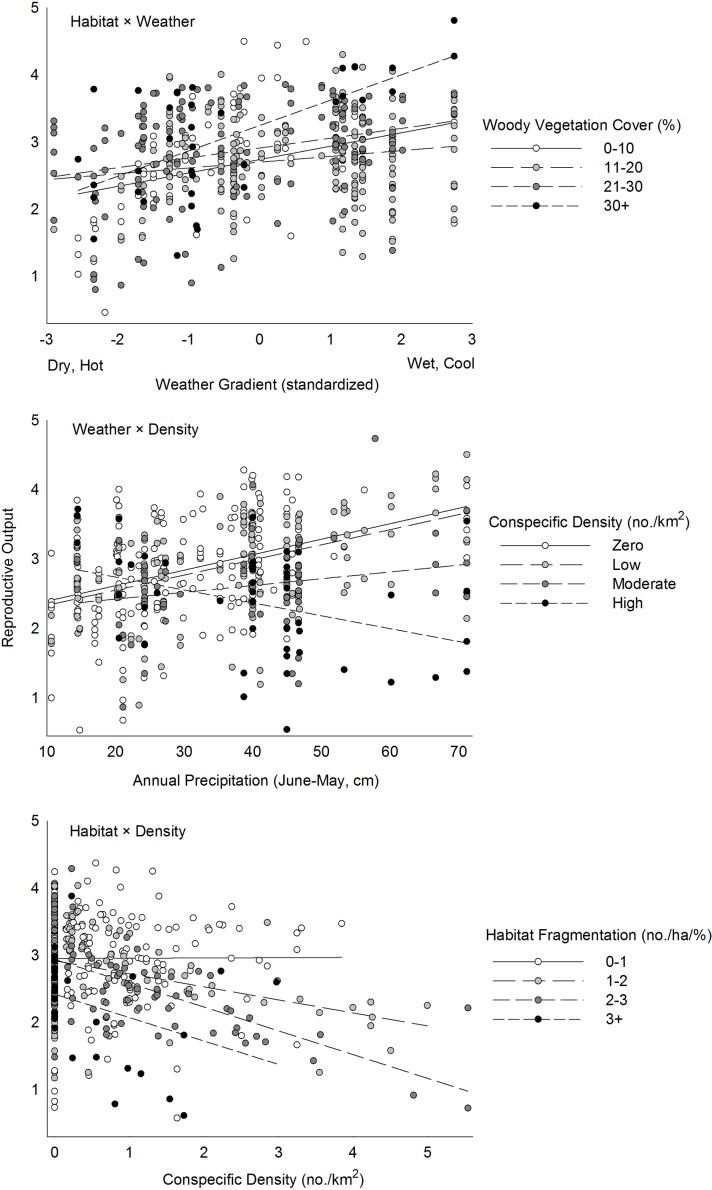
Interactive effects of important factors associated with different environmental components of habitat quality on reproductive output of ferruginous pygmy-owls in northwest Mexico, 2001–2010. The hypothetical weather gradient in the top figure was standardized based on annual precipitation and mean maximum temperature during the brooding season so as to represent conditions that ranged from wet and cool to hot and dry. Estimates are based on the top-ranked models that included these interactions in Table 6.

Models with interactions between habitat and weather, weather and conspecifics, and habitat and conspecifics all had greater support than corresponding additive models, but relative support for models with interactions between different components was similar (Table 6). The best model included interactions between T, P, and amount of woodland habitat (S5 Appendix). When this effect was evaluated across a hypothetical weather gradient ranging from favorable cool wet to harsh hot dry conditions, patches with more woodland amplified the positive effects of favorable weather more than those with less woodland (e.g., slopes varied; Fig. 8). Patches with more woodland, however, did not buffer the negative effects of harsh weather more than those with less woodland (e.g., intercepts did not vary). A highly competitive second-ranked model included an interaction between P and conspecific density (S5 Appendix). When this effect was evaluated across observed variation in P, R increased steadily with P when conspecifics were absent or present at low densities but less so at moderate densities (Fig. 8). When densities were high, however, R declined with increasing P, suggesting intraspecific competition offset the benefits of favorable weather.

**Fig 9:**
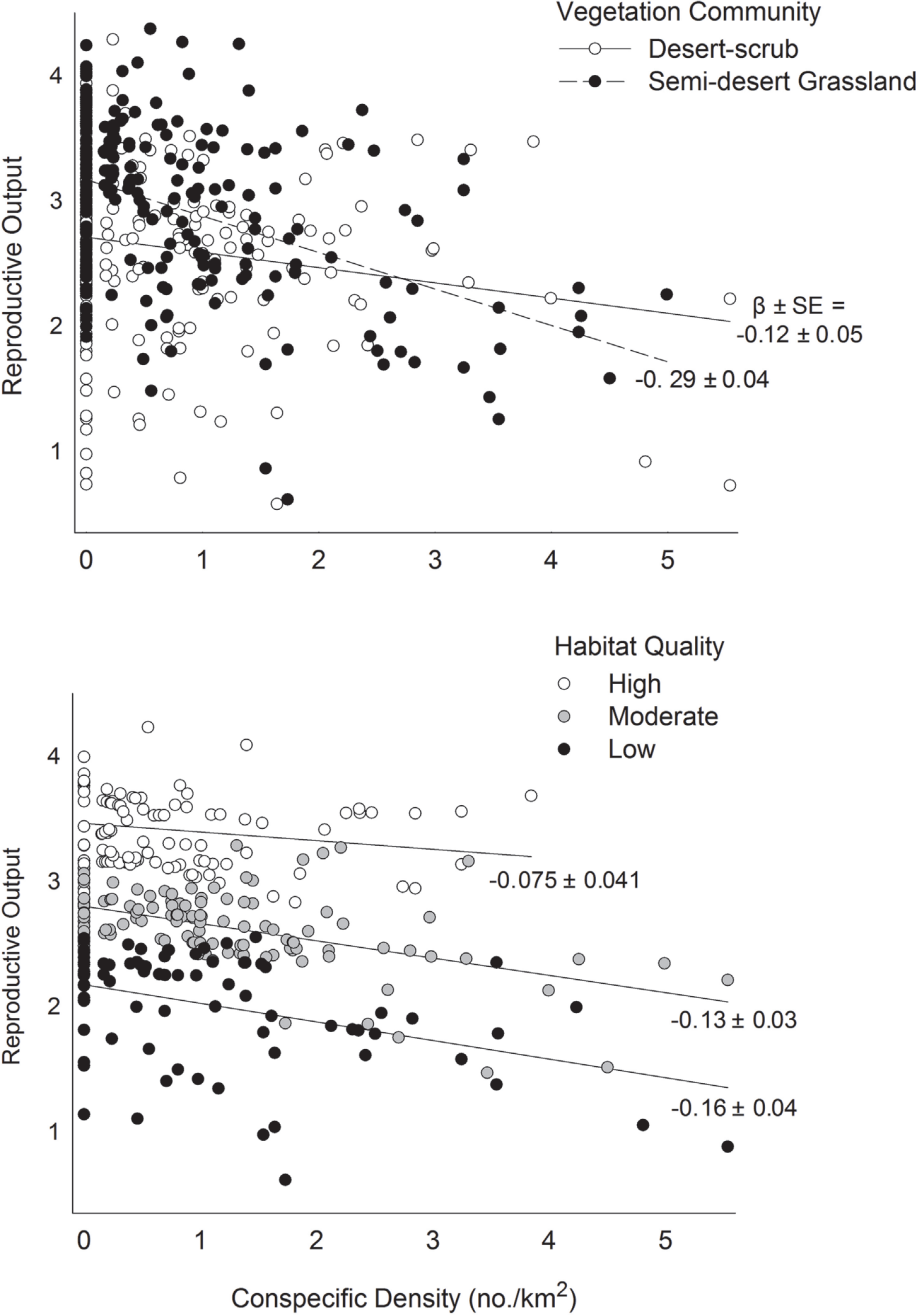
Effects of vegetation community and fundamnetal habitat quality on density-dependent declines in reproductive output of ferruginous pygmy-owls in northwest Mexico, 2001–2010. Habitat quality was classified as high (>3.0), moderate (>2.4–3.0), or low (0.9–2.4) based on patch-specific predictions of reproductive output from model 3 in Table 3. Slope parameters and SE are from least-squares regression. Estimates are based on the model {Habitat × Density + Weather} in Table 6 and S5 Appendix.

A model with interactions between habitat and conspecifics included interactions between density and three habitat factors (S5 Appendix). When the effect of woodland fragmentation was evaluated across observed variation in density, conspecifics had no effect on R when fragmentation was low, but R declined at increasing rates as fragmentation increased (Fig. 8). The effect of conspecifics also varied between macrohabitats; although fundamental habitat quality was higher on average in grasslands (e.g., greater intercept), R declined with increasing density at a rate 2.4 times greater in grasslands than in desert-scrub (Fig. 9). When the effects of all important habitat factors were considered, however, magnitudes of density-dependence varied with fundamental habitat quality and higher quality habitat buffered the negative effects of conspecifics more that lower quality habitat (Fig. 9).

## Discussion

We assessed habitat quality for ferruginous pygmy-owls at the scale of individual territory patches by estimating magnitudes of spatial and temporal variation in reproductive output (R), and assessing the relative contribution and specific effects of factors associated with three general components of the environment. Although factors associated with each component had important effects, habitat resources were much more influential than conspecifics or temporal factors such as weather. Nonetheless, temporal factors had marked effects in some years and the effects of important factors associated with each environmental component often interacted. Such patterns indicate that the environment can affect habitat quality in complex ways and that considering only one component in isolation of others may produce misleading results.

### Habitat

Habitat determines the availability of resources such as food and nest sites, the abundance of conspecific and heterospecific competitors, and vulnerability to predation, parasitism, and physiological stress [38,67]. Vegetation structure is a fundamental attribute of habitat because it simultaneously affects all of those factors [11,68]. In our system, attributes of vegetation structure likely reduce vulnerability to heterospecific enemies, promote foraging opportunities, and mitigate environmental harshness. With regard to vegetation structure, amount of woody vegetation cover had greater effects on performance than edge or woodland interior. Those patterns conform to general descriptions of habitat from across the range of pygmy-owls, which occur in a broad range of vegetation types that often include patches of dense vegetation interspersed with openings [69]. In our region, habitat included desert-scrub or tree-invaded grasslands near riparian woodlands with at least one saguaro with a suitable nest cavity.

Energy is a fundamental resource and thus differences in habitat quality should be linked to spatiotemporal variation in trophic energy [70,71]. Although woody vegetation cover best described R, differences in net primary productivity, indexed by normalized difference vegetation index (NDVI), provided a highly competitive explanation of the data. In fact, when each effect was compared, R increased more at high levels of NDVI but decreased more at low levels of vegetation cover. Those patterns are likely because NDVI varies with productivity of both woody and non-woody vegetation, which is rarely used directly by owls but provides important resources for prey, and because woody vegetation directly affects foraging space and abundance of heterospecific enemies. Because NDVI is closely linked to trophic energy, it has proven useful in explaining patterns of animal distribution, abundance, growth, and phenology, but associations between NDVI and vital rates are less common [58,72,73].

Nest-site availability and specific nest-cavity features can have large effects on abundance and demography of cavity-nesting birds [74,75]. In our system, important nest-cavity features include cavity height, entrance area, and orientation, which affect thermal conditions and predation risk and thus influence nest-site selection and its demographic consequences [76]. Because availability of optimal cavities increases with saguaro abundance [76], and because higher abundance of potential nests augments predator search time and reduces predator efficiency [77], patches with more saguaros provide higher quality habitat. Moreover, in the Sonoran Desert, pygmy-owls coexist with a diverse group of cavity nesters such as western screech-owl, American kestrel, elf owl (*Micrathene whitneyi*), gilded flicker (*Colaptes chrysoides)*, Gila woodpecker (*Melanerpes uropygialis*), and flycatchers (*Myarchis* sp.). When nest sites are rare, space use by those species becomes more concentrated, which likely increases cues to predators and promotes interactions with heterospecific competitors. Those and other processes (e.g., [78]) explain why habitat quality is lower in patches with fewer potential nests.

In arid environments, tree cover is often limited by soil moisture and woodlands are restricted to riparian areas that provide important foraging space and cover [79]. Although riparian areas used by owls rarely supported broadleaf trees, microphyllous trees such as mesquite provide important habitat for owls and prey [80,81]. In the Sonoran Desert, abundance and diversity of common prey often increases with vegetation cover, heterogeneity, and mesquite abundance, which are all associated with riparian areas and their ecotones [82–84]. Moreover, independent of the amount of woody vegetation cover, R declined with increasing woodland fragmentation, especially in grasslands where vegetation physiognomy and composition are less diverse and edge effects are likely higher. Thus, larger riparian woodlands provide higher quality habitat, which promotes the persistence of populations over time [50].

Food availability and predation risk are important drivers of reproduction in birds but their relative importance has been debated for decades [11,85,86]. Behavioral studies show that individuals balance the benefits of foraging with the predation risk incurred while doing so [87], and experimental studies show that augmenting food and reducing predation risk can have multiplicative benefits ([88,89], but see [90]). We found that the effect of a resource that mediates vulnerability to heterospecific enemies (nest-site abundance) depended on factors linked to food and foraging space (vegetation cover). For example, R increased with nest-site abundance at greater rates as vegetation cover increased, and prevalence of interactions with heterospecific enemies increased as nest-site abundance declined. Thus, once nest sites became sufficiently abundant to mitigate the effects of heterospecific enemies, the benefits of food and foraging space were realized. Where nests were rare, however, increasing vegetation cover had negative effects on R, likely because abundance of heterospecific enemies increases with vegetation cover [91], which was much higher in patches with few nest sites where we observed heterospecific interactions. Although our results suggest the effects of heterospecifics are greater than food, such effects can be challenging to separate because vegetation often simultaneously affects nesting and food resources [92,93]. Because in our system cavity abundance likely has little effect on food, our results provide strong evidence of interactive effects of predation and food on performance.

Few studies have assessed the relative ability of macrohabitat (e.g., vegetation community) vs. microhabitat (e.g., nest sites) factors to explain variation in animal abundance or demography [94,95], and most studies consider only macrohabitat [15,19,46,51,56,96,97] vs. both types of factors [5,52,98,99]. Because the spatial extent of macrohabitats are typically broad, studies that focus on them often pool observations of individuals within each macrohabitat rather than assess the effects of microhabitat at individual scales (but see [5,15,99]). Thus, in evaluating the patterns and drivers of variation in demography, studies focused on macrohabitats often treat populations within them as single demographic units and assume macrohabitat factors drive variation. We found that macrohabitat, microhabitat, and landscape (fragmentation) factors all explained variation in R among territories. Moreover, abundance of important resources often varied markedly in nearby patches in the same macrohabitat. Thus, had we considered only macrohabitat effects, important insights on processes that drove habitat quality would have been lost. Although habitat quality was higher on average in grasslands, variation in important microhabitat factors did not explain those differences and macrohabitat effects seemed to be driven by less extreme climates in grasslands. Assessments of habitat quality should consider macrohabitat, microhabitat, and potentially landscape factors because they may all be important and because macrohabitat effects could be driven by underlying variation in resources at smaller scales. Because habitat quality depends on the effects of resources and conditions on individual performance, which can vary continuously among territories in the same macrohabitat, evaluating habitat quality at this scale will provide more process-oriented insights.

### Weather

Temporal variation in weather can have indirect effects on vital rates by affecting resources or direct physiological effects [18]. In arid regions where climate is already harsh, extreme events can have major impacts on performance that may be increasing due to climate change [60,100]. Extreme events and the ecological crunches and bonanzas they create are characterized by major perturbations in vital rates that affect population dynamics [19,60] and potentially microevolution [101]. In the Sonoran Desert, increasing annual precipitation had largely positive effects on R, high brooding-season temperature had largely negative effects, and those same weather factors explained 75% of variation in population dynamics over an overlapping time period [50]. Moreover, a combination of hot dry conditions contributed to an apparent ecological crunch characterized by very low R. During such extremes, however, most owls still attempted to breed despite realizing lower performance.

In arid environments, precipitation often drives rapid increases in plant and insect biomass, which augments productivity of small vertebrates and then predator abundance [60,102]. Despite the importance of precipitation in this and other arid systems, precipitation had little effect on R when brooding-season temperatures were low. This pattern is likely due to indirect effects of precipitation on prey abundance and direct effects of temperature on prey activity during periods of rapid nestling growth and high energy demand. Activity levels of lizards depend on thermoregulatory requirements that vary with the physiology and behavior of individual species [103]. Because activity levels of common lizard prey decline at high temperatures (unpublished data), temperature likely also affects prey availability. When precipitation and thus prey abundance are high, however, temperature effects on prey availability are likely less important, especially in patches where prey diversity is high. When temperature is low and thermal conditions favorable, however, lizards likely remain active for longer periods, which increases prey availability and compensates for lower prey abundance. Interactive effects of temperature and precipitation on animal performance are rarely noted, potentially because they are rarely considered [104]. When precipitation affects food supply and temperature affects prey activity, however, such relationships may be common and have alarming implications given recent declines of owl populations [50] and predictions for increasing drought and higher temperatures linked to climate change [105,106].

### Conspecifics

Reproductive output within territory patches declined with increasing conspecific density at local scales around each focal patch. Thus, although pygmy-owls are territorial, conspecifics affect individual performance and this system does not conform strictly to an Ideal Despotic Distribution (IDD). Nonetheless, broad spatial heterogeneity in R among territories remained indicating general conformance to an IDD. Moreover, the effects of conspecifics were too weak to eliminate differences in realized performance among individuals, as has been observed in other despotic systems [31]. Such density-dependent declines in R at individual scales suggest interference or scramble competition, which are fundamental mechanisms of the Ideal Free Distribution [30]. Thus, our results add to a small but growing literature indicating such forms of competition also operate in despotic systems [29,31,107–109]. Because ideal distributions were developed to represent theoretical extremes, such mixed models may be common in nature and suggest a model of the IDD that includes interference should be developed.

Studies of density-dependent reproduction or survival often focus on population regulation or dynamics rather than habitat quality, and thus, are framed at population not individual scales [11,36]. Nonetheless, processes that create density dependence are not driven by the abundance of animals but rather by their effects on resources and social conditions. Here, we observed negative density dependence by measuring local conspecific densities around focal territories, but not at larger scales. Hence, the spatial scale at which density dependence is assessed can affect whether it is detected, which is why studies framed at scales larger than the spatial use of individual animals often fail to detect density dependence [62,110,111]. In our system, areas between some patches were occasionally occupied by intervening pairs of owls, which augmented local densities. As distances between neighbors contract, territory sizes and resource availability also contract and antagonistic interactions and costs of territorial defense increase, which are mechanisms that drive density dependence [25–27,112]. When density dependence is driven by interference or scramble competition, individual-specific metrics such as local density [62] or other distances-based metrics [109,113] are best suited to detect it.

In addition to interference, density dependence may also be driven by the effects of local interactions manifested at larger scales. This is because when habitat quality varies spatially and despots relegate subordinates to patches of lower quality through contest competition, increased variation in resource holding potential among individuals can cause average per capita performance to decline with population size [114,115]. After the effects of conspecifics at local scales were considered, however, R actually increased somewhat with regional population sizes. This pattern was likely driven by favorable weather augmenting food supply and carrying capacity, processes that complicate detecting density dependence at larger scales [11]. Regardless, to assess density-dependent habitat quality, estimating the effects of conspecifics at local scales relevant to individuals is essential.

### Relative contribution of components

Few studies have compared variation in vital or population growth rates across both space and time, especially at small scales relevant to individual animals [5,55,116]. We found that spatial process variation in R among territory patches was nearly 6 times greater than that across time, and that coefficients of process variation were 2.5 times greater across space than time, which suggests large habitat effects. In comparison, magnitudes of spatial vs. temporal process variation in R among spotted owl territories were nearly equal, a coefficient of spatial process variation was similar, and a coefficient of temporal process variation was much greater than in our system [5]. Thus, whereas habitat effects were also large, R was much more resilient to extreme events in our system. Coefficients of temporal variation in R of barn owls (*Tyto alba*, 0.081; [117]) and multiparous ungulates (0.091–0.098; [34]) are similar to that reported here (0.070) despite differences in life history.

Spatial variation in habitat can have large and persistent effects on performance [5,12–15], but few studies have estimated those effects in wild animal populations while also considering conspecifics or stochastic factors. Although habitat resources explained much greater proportions of variation in R than weather or conspecifics, in some years R varied by up to 56% due to weather and by up to 27% due to conspecifics. Thus, while good territories tended to remain good over time, the effects of conspecifics and weather reordered the realized quality of habitat across time. Although habitat effects should be strong in systems where individuals maintain exclusive use of space and depend on resources such as gross vegetation structure that are fairly static in time, stochastic and density-dependent processes can have large effects on vital and population growth rates, and should be considered when estimating habitat quality [11,17,118].

### Interactions among components

In evaluating how the environment affected habitat quality, we found that the effects of habitat resources, weather, and conspecifics interacted in complex and sometimes novel ways. When evaluated in a model selection framework, evidence for interactions among components was stronger than for additive relationships but relative support for interactions between different components was similar suggesting multiple processes influence habitat quality simultaneously. Van Horne et al. [19] suggested that when weather affects food supply, habitat quality is likely driven by interactions between vegetation and weather. Her assertion was based on observations of varying demographic responses to weather by squirrels in habitat of different characteristics, but since, few studies have addressed such relationships. Franklin et al. [5] found that high-quality habitat buffered the effects of harsh weather on survival but not reproduction of spotted owls. Here, we found that territories with greater vegetation cover magnified the benefits of favorable weather. High-quality habitat, however, failed to buffer the negative effects of harsh weather suggesting adverse conditions affected all individuals equally. Because in our system precipitation augments prey abundance that is likely already higher in areas with more vegetation cover, owls that occupy those areas attain multiplicative benefits when conditions are favorable, which further suggests interactions between habitat and weather are pervasive. Such patterns indicate the importance of considering broad temporal contexts when evaluating habitat quality and suggest caution when inferring differences in habitat quality based on short-term studies. If some habitat features are capable of buffering the negative effects of harsh weather, habitat quality could be higher where animals are more resilient to weather than in areas that occasionally support very high performance. Moreover, if some habitat features magnify the benefits of favorable weather, then relative differences in habitat quality may not be apparent until such conditions are present. Understanding the extent to which habitat mediates weather effects has important implications for management in a changing climate.

Despite a long history of debate, recognition that the effects of extrinsic factors can depend on conspecific densities has become widespread in recent decades [36,119]. The most frequently reported example of such patterns are in temperate systems and involve increasing negative effects of harsh winters as conspecific densities rise [34,35]. Here, in a Neotropical system, we found that the positive effects of favorable weather on R acted in a density-independent manner at low densities. When densities were high, however, R decreased even as weather conditions improved suggesting the positive effects of favorable weather were offset by intraspecific competition. Although interactions between weather and conspecific density are well documented during periods of resource scarcity [35,120], very few studies suggest the same during periods of resource abundance (e.g., [121]). This tendency is likely because key factors that drive performance vary geographically and because there are few studies in tropical and subtropical vs. temperate systems [11,34,36]. While broad generalizations have yet to fully emerge, density-dependent mortality in the non-growing season is likely more important in temperate vs. tropical systems, where density-dependent R in the growing season seems more influential [86,120,122]. Although we did not assess mortality in the non-growing season, winter severity has no effect on population dynamics in this system [50].

Few studies address how habitat resources mediate the effects of conspecifics on individual performance [32,33,96,99]. In ungulate systems, McLoughlin et al. [33] found that high-quality habitat had positive effects on lifetime reproductive success at low but not at high densities, and, Pettorelli et al. [96] found that juvenile survival was high regardless of habitat quality at low densities and that high-quality habitat buffered the negative effects of conspecifics at high densities. Here, we found that important habitat resources mediated the effects of conspecifics on R in different ways, and that habitat of higher fundamental quality buffered the negative effects of conspecifics more than low-quality habitat. Our findings are novel because we considered continuous variation in habitat quality based on the effects of microhabitat, macrohabitat, and landscape factors and because the effects of conspecifics varied depending on the habitat factors considered. With regard to landscape factors, conspecifics had no effect on R at low levels of woodland fragmentation but increasingly negative effects as fragmentation increased. With regard to macrohabitats, rates of negative density dependence were higher in grasslands despite the fact that grasslands provided higher quality habitat on average in the absence of conspecifics. Nonetheless, when the effects of all important factors were considered together, high-quality habitat buffered the negative effects of conspecifics more than low-quality habitat, which could be true in a broad range of systems. Consequently, had we considered only the effects of macrohabitats (e.g., [32]), insights regarding the effects of conspecifics would have varied. Although conspecifics may degrade realized habitat quality in a general sense, high-quality resources can buffer those effects and provide greater fitness rewards to occupants. While identifying mechanisms that drove these patterns was not our goal, we suspect territory sizes decline with increasing patch quality, which makes individuals in high-quality habitat generally less susceptible to changes in conspecific density.

### Implications

Assessments of habitat quality often focus solely on spatial variation in habitat resources. However, as we show, factors such as conspecific density and weather that vary both spatially and temporally can mediate habitat effects. In our system, individuals that occupied habitat of high fundamental quality performed better not only because resources were better, but also because those areas buffered the negative effects of conspecifics and amplified the benefits of favorable weather. The effects of weather and conspecifics, however, reordered the relative quality of territories over time, patterns likely to be more extreme in systems where temporal variation and conspecific densities are higher. Thus, although natural selection should promote the evolution of habitat selection based largely on spatial components, land managers should consider the effects of conspecifics and stochastic factors when estimating habitat quality and prioritizing areas for conservation, especially when assessments are made over the short term. Additionally, because we only measured R but habitat quality is a function of both R and survival, inferences based on territory-specific population growth rates could vary somewhat and future efforts should integrate survival (e.g., [5]).

Information on factors that drive habitat quality is important for guiding management, especially for pygmy-owls that have declined markedly in our region [48,50]. Although habitat quality is best measured at an individual scale, conservation focuses on populations. Thus, understanding how conspecifics affect individual performance and how resources and individuals are distributed is important for management. We found that conspecifics had only moderate effects on performance that declined as fundamental habitat quality increased. Because the negative effects of conspecifics were low at densities <0.5 territories/km^2^, small-scale efforts to improve habitat that matches this scale will be most efficient. Although we did not assess how resources affected territory size or density at larger scales, high-quality territories were often adjacent to those of low quality, and populations did not conform to an Ideal Free Distribution. Thus, strategies focused on enhancing habitat resources that directly affect performance will be more efficient for conservation than those focused on owl density [6], especially when they simultaneously augment habitat area.

Our results suggest a variety of strategies for bolstering recovery prospects. Abundance of potential nest cavities had strong positive effects on performance, especially in areas with high woody vegetation cover. Thus, management that promotes the survival and recruitment of saguaros will benefit owls. Additionally, augmenting cavity abundance by erecting nest boxes or translocating saguaros will enhance habitat quality, especially when guided by recommendations on cavity features [76] and focused in areas that support large unfragmented woodlands.

Most historical records of pygmy-owls in the Sonoran Desert were from large riparian areas in valley bottoms that have been lost or degraded in the last century [48]. Restoring these once extensive desert riparian areas by fostering establishment and growth of mesquite and other trees will enhance recovery prospects for pygmy-owls while also creating habitat for other species. Moreover, because increasing woody vegetation cover amplified the positive effects of favorable weather, and lower woodland fragmentation reduced the negative effects of conspecifics, restoring large unfragmented woodlands in valley bottoms where they have been lost or degraded should have multiplicative benefits, especially in more arid regions.

Hot and dry conditions had negative effects on reproduction regardless of vegetation. Thus, enhancing habitat quality may not be a realistic strategy for confronting climate change unless habitat resources buffer the effects of harsh weather on survival (e.g., [5]). Future research in this and other systems should assess the degree to which high-quality resources mediate the effects of harsh weather on survival, and identify resources that promote persistence in the face of climate change. More generally, because the collective environment affects habitat quality in complex ways, integrative approaches that consider habitat, stochastic factors, and conspecifics are needed to guide management.

## Supporting Information

S1 Appendix Models representing the hypothesized effects of spatial factors on reproductive output of ferruginous pygmy-owls in northwest Mexico, 2001–2010. (PDF)

S2 Appendix Models representing hypothesized effects of temporal factors on reproductive output of ferruginous pygmy-owls in northwest Mexico, 2001–2010. (PDF)

S3 Appendix Description of remote sensing methods used to quantify woody vegetation cover and other land-cover classifications within territory patches of ferruginous pygmy-owls in northwest Mexico, 2001–2010. (PDF)

S4 Appendix Factors, spatial scales, and models that described the effects of presence and abundance of conspecifics on reproductive output of ferruginous pygmy-owls in northwest Mexico, 2001–2010. (PDF)

S5 Appendix Models and estimates of the interactive effects of spatial, temporal, and conspecific factors on reproductive output of ferruginous pygmy-owls in northwest Mexico, 2001–2010. (PDF)
