# Correction: Spatial, Temporal, and Density-Dependent Components of Habitat Quality for a Desert Owl

**DOI:** 10.1371/journal.pone.0141178

**Published:** 2015-10-20

**Authors:** Aaron D. Flesch, Richard L. Hutto, Willem J. D. van Leeuwen, Kyle Hartfield, Sky Jacobs

The captions for Figs 2 and 3 are incorrectly switched. The caption for Fig 3 should be the caption for Fig 2, and the caption for Fig 3 should be the caption for Fig 2. Please see the corrected captions here.

**Fig 2:**
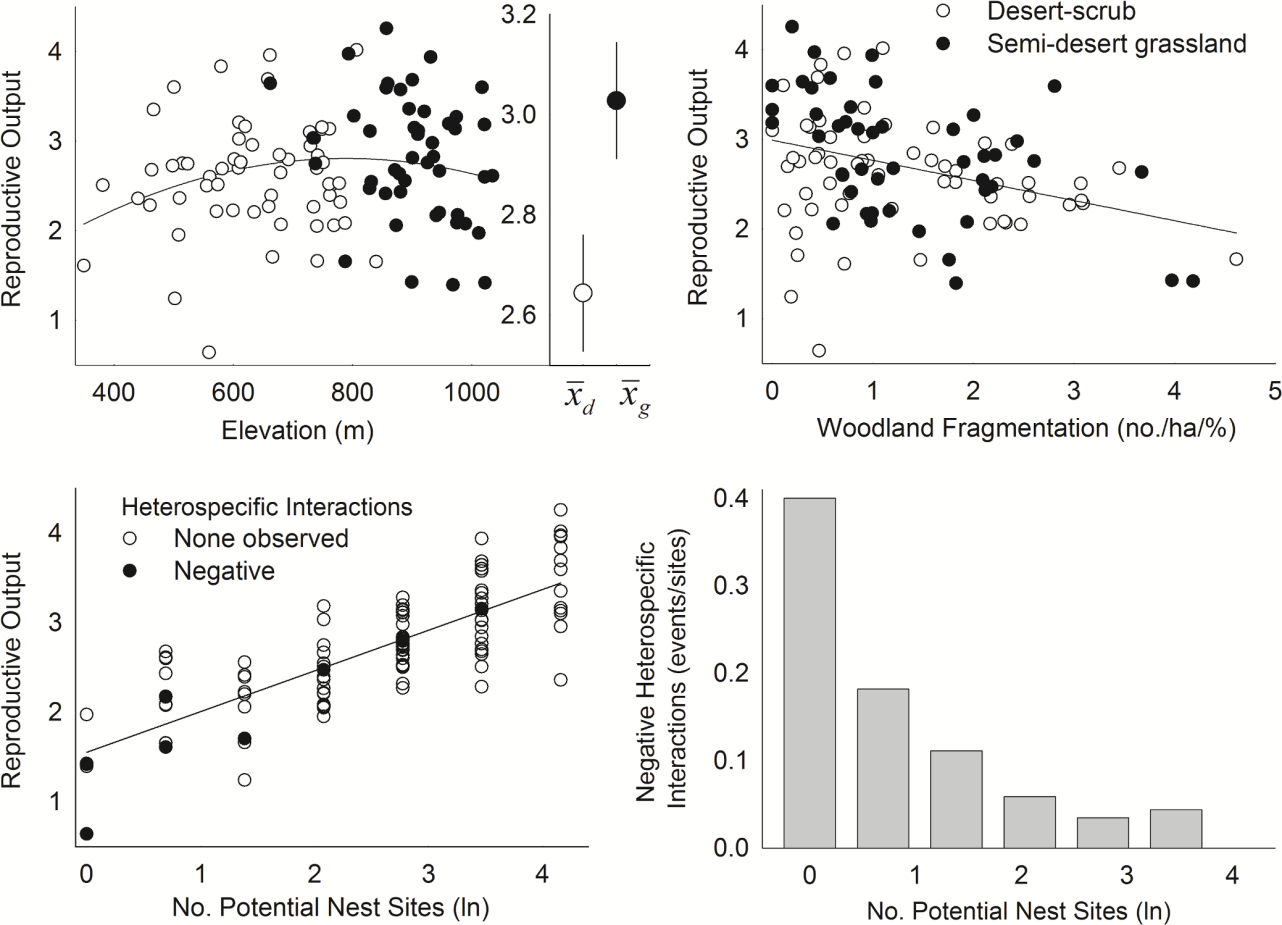
Effect of habitat factors on reproductive output of ferruginous pygmy-owls in northwest Mexico, 2001–2010. Lower right figure shows the number of negative heterospecific interactions observed divided by the total number of territory patches in each group across a gradient of increasing abundance of potential nest sites. Filled circles in upper figures are patches in semi-desert grasslands whereas those in the lower figure are patches where we observed negative heterospecific interactions. Estimates of reproductive output are based on model 3 in Table 3. Inset in upper left figure shows means (± SE) in each vegetation community.

**Fig 3:**
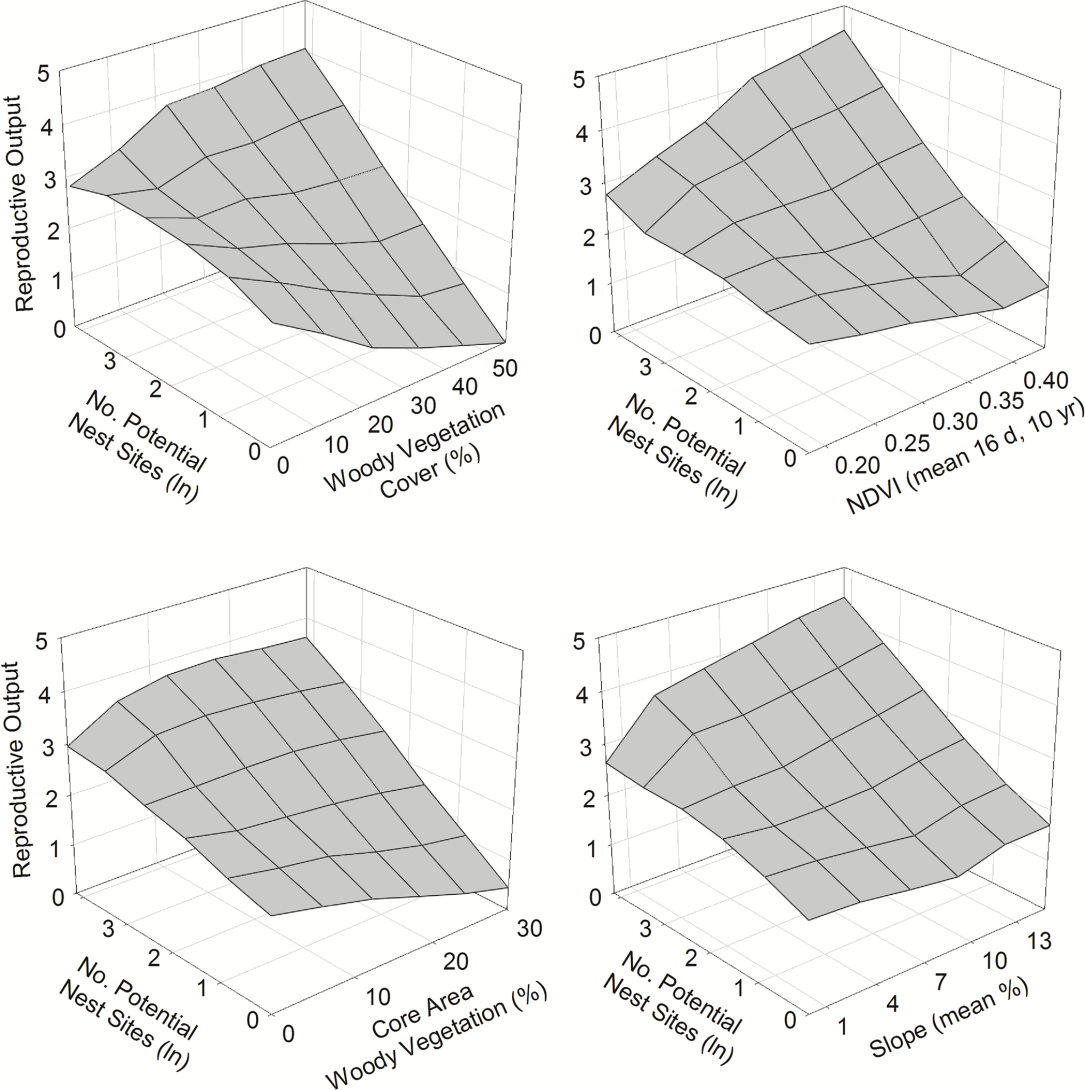
Interactive effects of abundance of potential nest sites and other habitat factors on reproductive output of ferruginous pygmy-owls in northwest Mexico, 2001–2010. Estimates of reproductive output are based on the top-ranked models that include each of the habitat factors represented as summarized in Table 3.
