# Supplementary material for: Spatial, Temporal, and Density-Dependent Components of Habitat Quality for a Desert Owl
Source: PLoS One. 2015 Mar 18;10(3):e0119986. doi: 10.1371/journal.pone.0119986 (PMC4364994; doi:10.1371/journal.pone.0119986)
Supplement: S1 Appendix — (PDF) [file pone.0119986.s001.pdf]

S1 Appendix: Description of spatial hypotheses.

Table S1A. Models representing the hypothesized effects of spatial habitat factors on reproductive output of ferruginous pygmy-owls in northern Sonora, Mexico, 2001-2010. Rationale for hypotheses is described in the text. Model B was a base model and included effects considered in all models.

	Model	Hypothesis	Expected results
B)	Cavities, Elevation, Vegetation community	Nest-site availability and environmental harshness explains R	Positive effect of Cav, positive or quadratic effect of Elev, positive effect of semi-desert grassland
1)	Woodland, Topography	Amount of woodland habitat and topography diversity explains R	Positive or quadratic effect of Hab and Topography or interaction between Cav and Hab and/or Cav and Topography
2)	Woodland, Topography, Disturbance	Amount of woodland habitat, disturbance, topography diversity explains R	Positive or quadratic effect of Hab and Topography or interaction between Cav and Hab and Cav and Topography negative effect of Disturbance
3)	Woodland, Fragmentation	Amount and fragmentation of woodland habitat, disturbance, topography diversity explains R	Positive or quadratic effect of Hab and Topography or interaction between Cav and Hab and Cav and Topography negative effect of Disturbance, negative effect of Fragmentation
4)	Woodland, Topography, Fragmentation	Amount and fragmentation of woodland habitat, topography diversity explains R	Positive or quadratic effect of Hab and Topography or interaction between Cav and Hab and Cav and Topography, negative effect of Fragmentation
5)	Core-Area Woodland	Amount of core habitat explains R	Positive or quadratic effect of Core or interaction between Cav and Core
6)	Core-Area Woodland, Topography	Amount of core habitat and topography diversity explains R	Positive or quadratic effect of Core and Topography or interaction between Cav and Core area and Cav and Topography

7)	Core-Area Woodland, Topography, Disturbance	Amount of core habitat, disturbance, and topography diversity explains R	Positive or quadratic effect of Core and Topography or interaction between Cav and Core and Cav and Topography, negative effect of Disturbance
8)	Edge	Amount of edge explains R	Positive or quadratic effect of Edge or interaction between Cav and Edge
9)	Edge, Topography	Amount of edge and topography diversity explains R	Positive or quadratic effect of Edge and Topography or interaction between Cav and Edge and Cav and Topography
10)	Edge, Topography, Disturbance	Amount of edge, disturbance, and topography diversity explains R	Positive or quadratic effect of Edge and Topography or interaction between Cav and Edge and Cav and Topography, negative effect of Disturbance
11)	Primary Productivity	Primary productivity explains R	Positive or quadratic effect of NDVI or interaction between Cav and NDVI

Notes: positive and negative effects were assessed with both linear and pseudo-threshold ln(x +1) forms of covariates. Each hypothesis was represented by a suite of models that each considered a different related covariate within each variable group in Table 1, and linear, pseudo-threshold, and quadratic forms of covariates.
