# Supplementary material for: Spatial, Temporal, and Density-Dependent Components of Habitat Quality for a Desert Owl
Source: PLoS One. 2015 Mar 18;10(3):e0119986. doi: 10.1371/journal.pone.0119986 (PMC4364994; doi:10.1371/journal.pone.0119986)
Supplement: S2 Appendix — (PDF) [file pone.0119986.s002.pdf]

S2 Appendix: Description of temporal hypotheses.

Table S2A: Models representing the hypothesized effects of weather, primary productivity, and seasonal timing of peak primary productivity on reproductive output of ferruginous pygmy-owls in northern Sonora, Mexico, 2001-2010. Rationale for hypotheses is described in the text.

Model	Hypothesis	Expected results
1) Winter Temperature	Low temperatures during winter stress period explains R	Positive effect of T
2) Nesting Temperature	High temperatures during nestling stress period explains R	Negative effect of T
3) Precipitation	Precipitation before nesting explains R	Positive or quadratic effect of P
4) Productivity	Primary productivity before nesting explains R	Positive or quadratic effect of NDVI
5) Timing	Timing of peak productivity before nesting explains R	Negative or quadratic effect of S_{NDVI}
6) Nesting Temperature Precipitation	High temperatures during and precipitation before nesting explains R	Negative effect of T, positive or quadratic effect of P, or interaction between T and P
7) Nesting Temperature, Productivity	High temperatures during and primary productivity before nesting explains R	Negative effect of T, positive or quadratic effect of NDVI, or interaction between T and NDVI
8) Nesting Temperature, Precipitation, Productivity	High temperatures during and both precipitation and primary productivity before nesting explains R	Negative effect of T, positive or quadratic effect of P and NDVI, or interaction between T and P and P and NDVI

9)	Nesting Temperature, Precipitation, Timing	High temperatures during and both precipitation and timing of productivity before nesting explains R	Negative effect of T, positive or quadratic effect of P, negative or quadratic effect of S_{NDVI}, or interactions
10)	Nesting Temperature, Productivity, Timing	High temperatures during and both primary productivity and timing of productivity before nesting explains R	Negative effect of T, positive or quadratic effect of NDVI, negative or quadratic effect of S_{NDVI}, or interactions
11)	Nesting Temperature, Timing, Precipitation, Productivity	High temperatures and precipitation, productivity, and timing of productivity before nesting explains R	Negative effect of T, positive or quadratic effect of P and NDVI, negative or quadratic effect of S_{NDVI}, or interactions
12)	Precipitation, Productivity	Precipitation and primary productivity before nesting explains R	Positive or quadratic effect of P and NDVI, or interaction between P and NDVI
13)	Precipitation, Timing	Precipitation and timing of peak productivity before nesting explains R	Positive or quadratic effect of P, negative or quadratic effect of S_{NDVI}, or interactions
14)	Precipitation, Productivity, Timing	Precipitation, primary productivity, and timing of productivity before nesting explains R	Positive or quadratic effect of P and NDVI, negative or quadratic effect of S_{NDVI}, or interactions
15)	Productivity, Timing	Primary productivity and timing of productivity before nesting explains R	Positive or quadratic effect of NDVI, negative or quadratic effect of S_{NDVI}, or interactions

Notes: positive and negative effects were assessed with both linear and pseudo-threshold ln(x +1) forms of covariates. Each hypothesis was represented by a suite of models that each considered a different related covariate within each variable group in Table 2, and linear, pseudo-threshold, and quadratic forms of covariates.
