# Supplementary material for: Spatial, Temporal, and Density-Dependent Components of Habitat Quality for a Desert Owl
Source: PLoS One. 2015 Mar 18;10(3):e0119986. doi: 10.1371/journal.pone.0119986 (PMC4364994; doi:10.1371/journal.pone.0119986)
Supplement: S3 Appendix — (PDF) [file pone.0119986.s003.pdf]

S3 Appendix. Description of multi-sensor remote sensing methods used to classify land cover and quantify cover of woody vegetation.

Remote Sensing Data Selection and Pre-Processing – The best time of year to create a Landsat based woody cover classification and estimation was determined by examining 250 meter 16-day composite Normalized Difference Vegetation Index (NDVI) time series data from the Moderate Resolution Imaging Spetroradiometer (MODIS) (Fig. S3A). A summer time period for which NDVI values were low was considered the best, in order to minimize the impact of the grass cover and avoid the chances of herbaceous vegetation being confused with tree cover. A few periods met the low vegetation signature criteria, but 2007 was chosen for mainly two reasons. The base value for NDVI in 2007 occurred in June which correlated well with cloudless 30 meter Landsat 5 Thematic Mapper (TM) image data. Another plus was that National Agricultural Imagery Program 1 meter

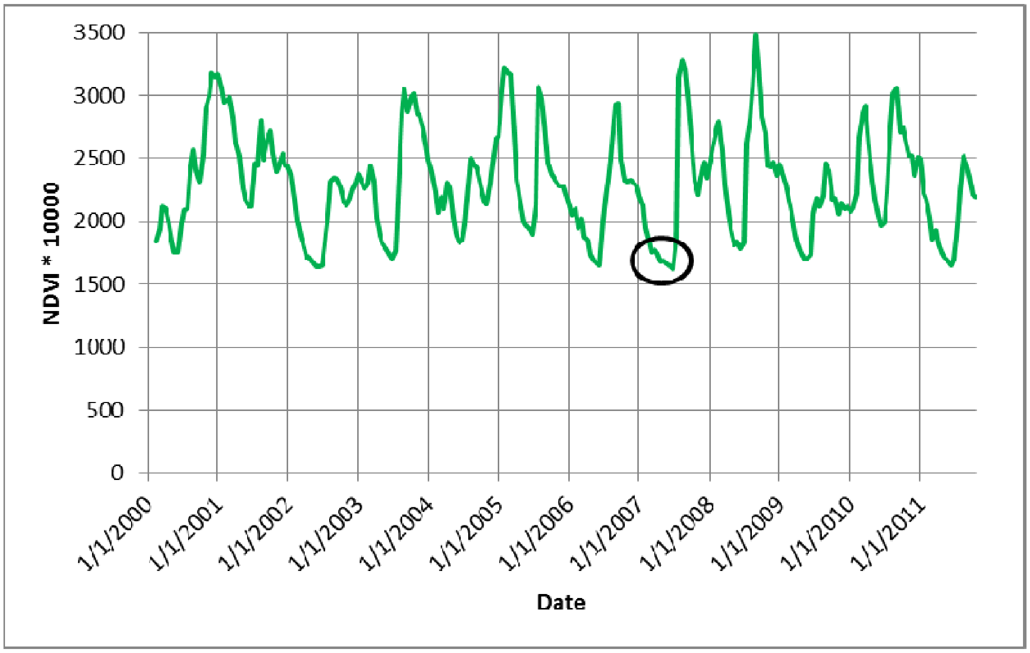

Fig. S3A: Example of multiyear MODIS time series data for one of the nest-sites, highlighting the baseline NDVI values during June of 2007.

data (NAIP) was flown for Arizona and parts of Mexico at the end of June 2007. The high resolution NAIP multispectral data allowed us to assess the accuracy of the woody cover product created with the coarser TM image data. Landsat image data were downloaded from: http://glovis.usgs.gov/. The study area was contained within four different Landsat images: Path 35 Row 39, Path 36 Row 38, Path 36 Row 39, and Path 37 Row 38. The majority of the nesting sites fell within Path 36 Row 38 and Path 36 Row 39 which were captured by the TM sensor on June 27 of 2007. The image for Path 35 Row 39 was captured on May 19, 2007, while the image for Path 37 Row 38 was captured on June 18, 2007.

The four images acquired were run through the Landsat Ecosystem Disturbance Adaptive Processing System (LEDAPS) model (Masek et al. 2006, Masek et al. 2012) to correct for the effects of atmosphere on the reflectance data. The four atmospherically corrected images were then stitched together to create one image of the study area. An NDVI image and a Principal Components Analysis (PCA) image were created from the multispectral image in order to reduce residual noise in the data and better identify woody cover in the region.

Land Cover Classification - A Classification and Regression Tree algorithm was applied to create the land cover classification for this semi-arid region, similar to Villeareal et al. (2012). Training data, to perform the Landsat land cover classification, was acquired from 1 meter multispectral NAIP and high spatial resolution Google Earth data. The NAIP data were collected on June 23 only four days before the majority of the Landsat data was captured making it ideal for training and assessment. Three classes

Table S3A: Accuracy assessment of Landsat land cover classification using spectral reflectance, NDVI, and PCA data.

Landcover Class		1	2	3	Total	User	Commission	Kappa
Woody Cover	1	49	0	1	50	98.00%	2.00%	0.97
Non-Woody Cover	2	0	50	0	50	100.00%	0.00%	1.00
Agriculture	3	1	0	49	50	98.00%	2.00%	0.97
	Total	50	50	50	150			
	Producer	98.00%	100.00%	98.00%		148		
	Omission	2.00%	0.00%	2.00%			98.67%	
	Kappa	0.97	1.00	0.97				0.98

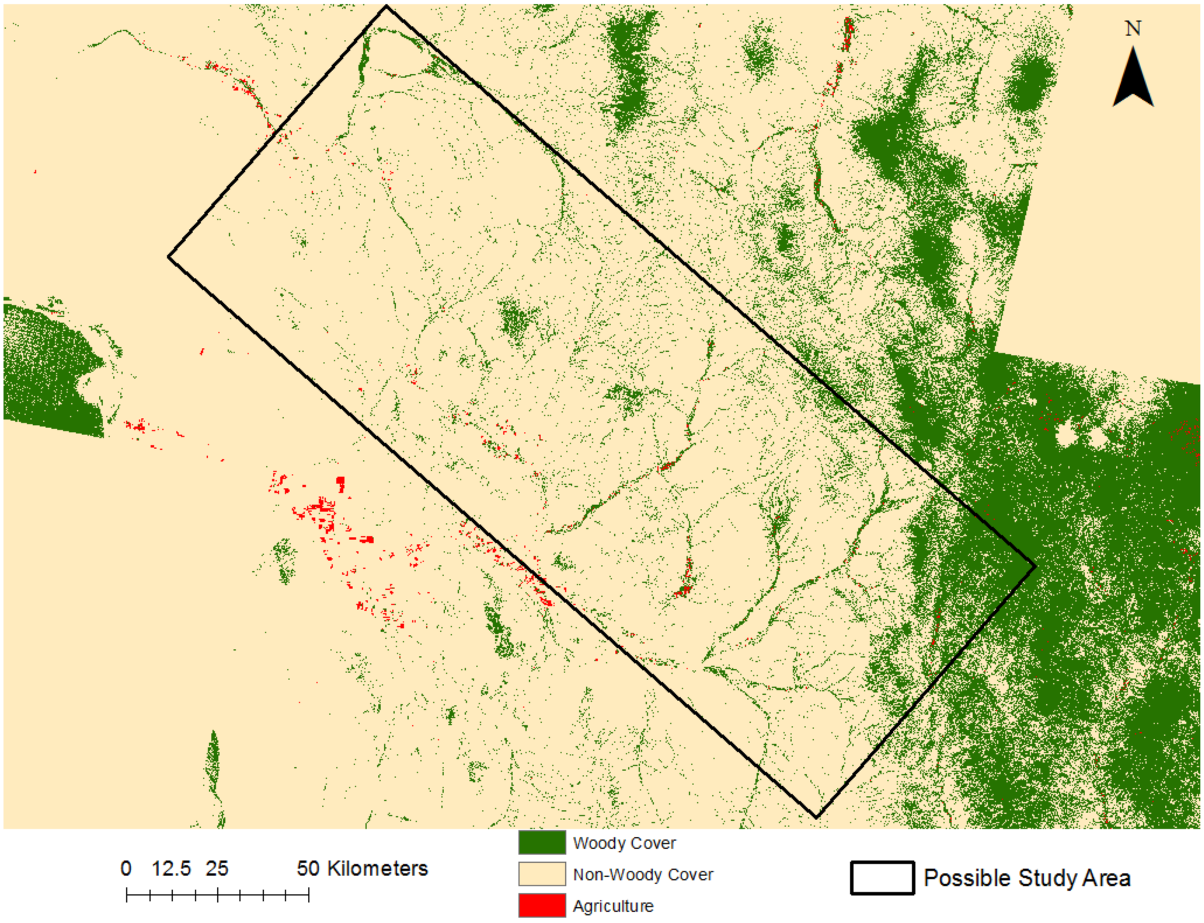

Fig. S3B: Land cover classification performed using Landsat spectral data, NDVI data, and a PCA image.

were trained upon: woody cover, non-woody cover, and agriculture. Points were selected for each of the classes taking into account NDVI values in order to help discriminate between classes. Fifty points were also collected for each of the classes in order to assess the accuracy of the land cover classification. The classification was run with CART using the spectral reflectance, NDVI, and PCA data, achieving an overall accuracy of about 99 percent (Table S3A; Fig. S3B).

Woody cover estimation - Using the NAIP data as reference, a Landsat pixel was selected as a representation of pure woody cover, while another pixel was selected as a representation of pure soil in order to perform a linear Spectral Mixture Analysis (SMA) (Van Leeuwen et al. 1997). The two pixels were selected based on visual interpretation of the NAIP data along with the spectral signatures of the selected Landsat pixels. The output from the SMA results in the fractional abundance of vegetation within each pixel (Fig. S3C).

The 30m vegetation abundance data were calibrated with classified woody cover data from 1m NAIP multispectral data. Using a 30 meter by 30 meter polygon grid a range of pixels were selected from the vegetation abundance raster representing the following abundance ranges: 0-0.1, 0.1-0.2, .2-.3, ,etc. Percent woody cover was then extracted from the NAIP land cover classification by taking a count of the number of pixels classified as woody cover within the 30 meter by 30 meter grid. This count could range from 0 to 900 so it was divided by 9 in order to get a percent cover. Percent woody cover was estimated based on the SMA vegetation abundance data for the pixels and their

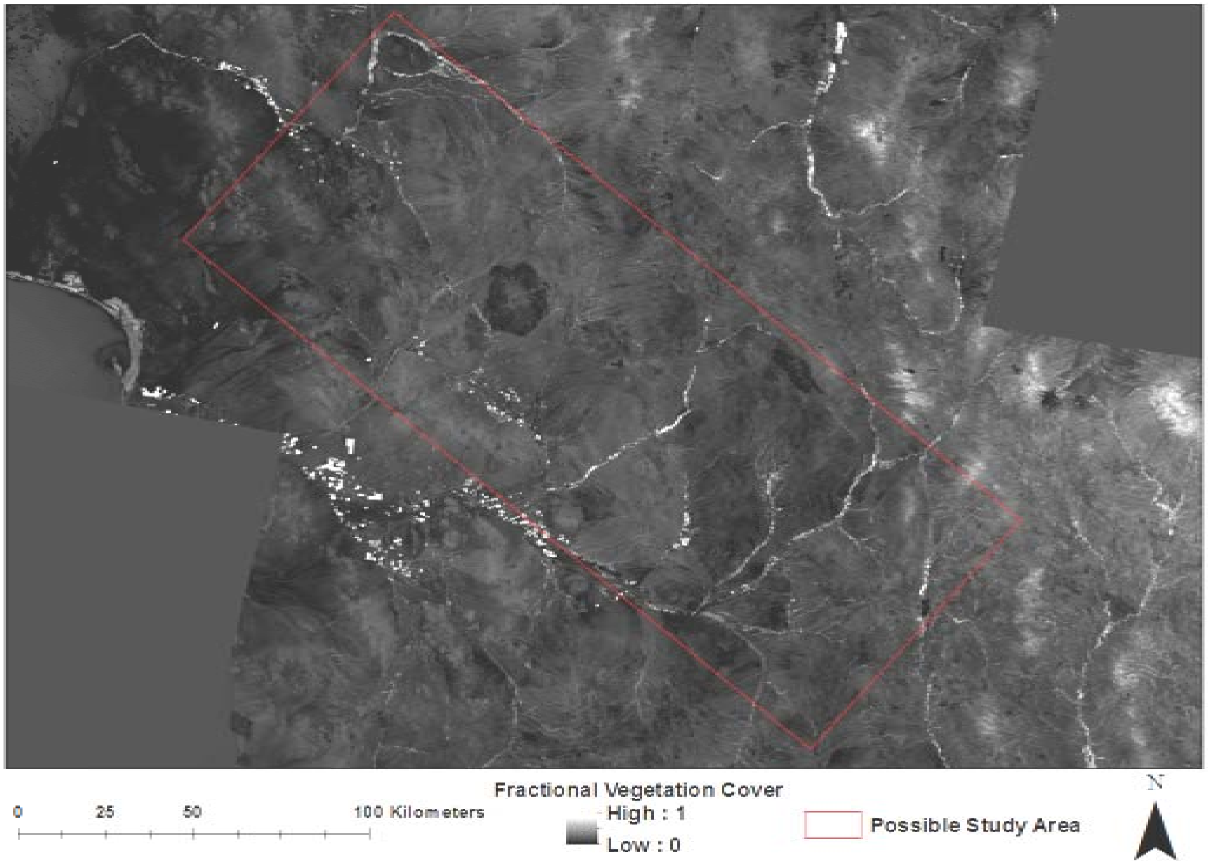

Fig. S3C: Linear spectral mixture model - vegetation abundance results for the Landsat mosaic for June 27, 2007. White represents high vegetation cover, while black represents low vegetation cover.

corresponding NAIP-based percent woody cover estimates. Using a linear regression, the relationship between the unmixed pixels and NAIP classification had an R^2 of 0.7881 (Fig. S3D). The equation in Figure A5 was applied to the vegetation abundance image to create a map of percent woody cover for the entire study area (Fig. S3E).

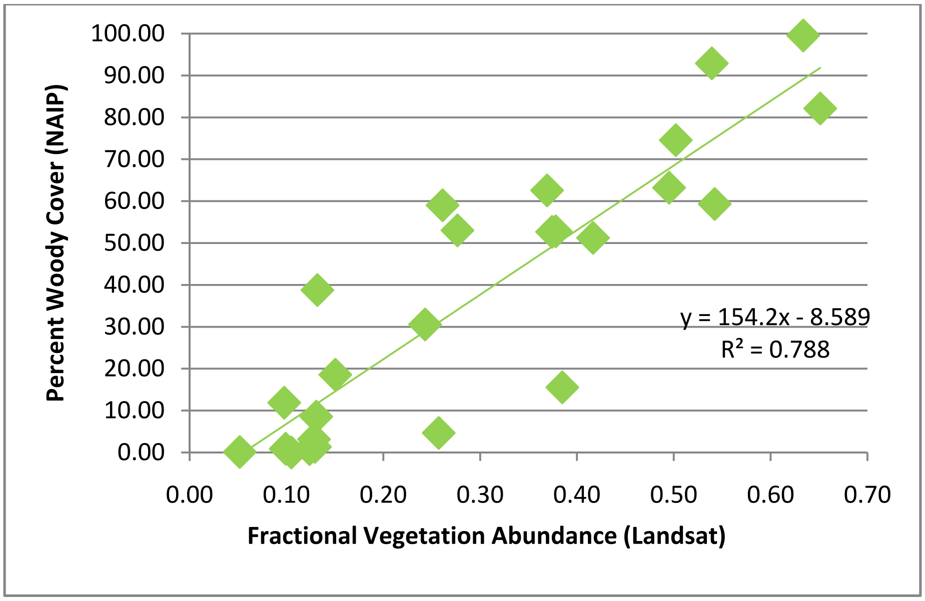

Fig. S3D: Results of linear regression of NAIP percent woody cover measurements and the Landsat-based vegetation abundance from the linear spectral mixture model.

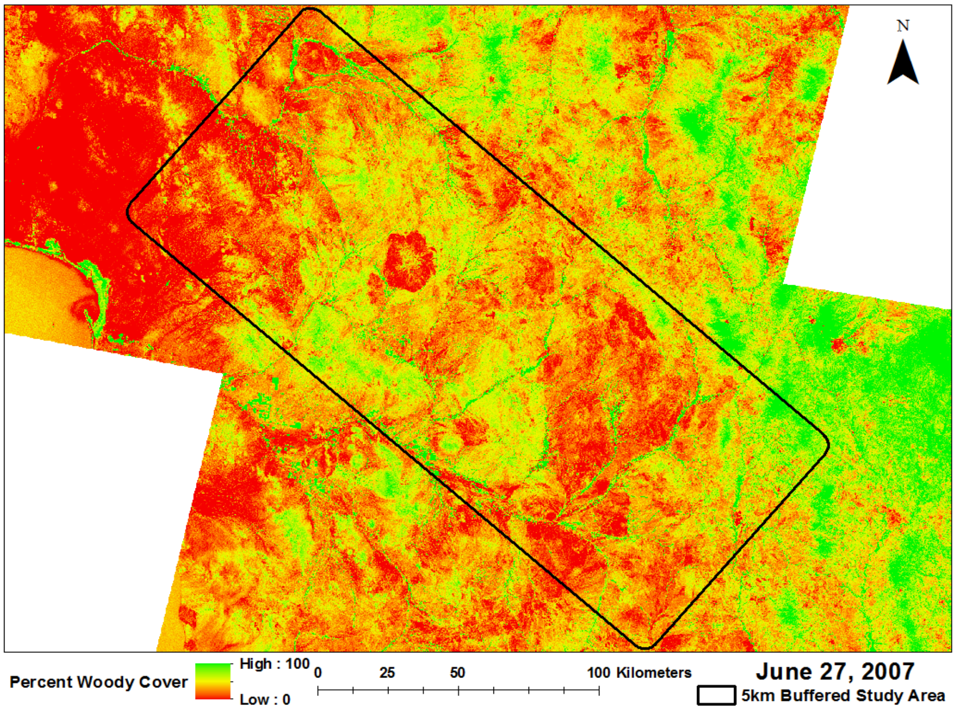

Fig. S3E: Percent woody cover image as a result of calibrating the vegetation abundance (LANDSAT) with NAIP-based woody cover data.

References

Masek, J. G., E.F. Vermote, N. Saleous, R. Wolfe, F.G. Hall, F. Huemmrich, F. Gao, J. Kutler, and T. K. Lim. 2012. LEDAPS Landsat Calibration, Reflectance, Atmospheric Correction Preprocessing Code. Model product. Available on-line [http://daac.ornl.gov] from Oak Ridge National Laboratory Distributed Active Archive Center, Oak Ridge, Tennessee, U.S.A. http://dx.doi.org/10.3334/ORNLDAAC/1080

Masek, J. G., E.F. Vermote, N. Saleous, R. Wolfe, F.G. Hall, F. Huemmrich, J. K. F. Gao, and , and T.K. Lim. 2006. A Landsat surface reflectance data set for North America, 1990-2000. Geoscience and Remote Sensing Letters 3:68-72.

McGarigal, K., S. A. Cushman, and E. Ene. 2013. FRAGSTATS v4: Spatial Pattern Analysis Program for Categorical and Continuous Maps. Computer software program produced by the authors at the University of Massachusetts Amherst.

Van Leeuwen, W. J. D., A. R. Huete, C. L. Walthall, S. D. Prince, A. Bégué, and J. L. Roujean. 1997. Deconvolution of remotely sensed spectral mixtures for retrieval of LAI, fAPAR and soil brightness. Journal of Hydrology 188-189:697-724.

Villarreal, M. L., W. J. D. van Leeuwen, and J. R. Romo-Leon. 2012. Mapping and monitoring riparian vegetation distribution, structure and composition with regression tree models and post-classification change metrics. International Journal of Remote Sensing 33:4266-4290.
