# Supplementary material for: Spatial, Temporal, and Density-Dependent Components of Habitat Quality for a Desert Owl
Source: PLoS One. 2015 Mar 18;10(3):e0119986. doi: 10.1371/journal.pone.0119986 (PMC4364994; doi:10.1371/journal.pone.0119986)
Supplement: S4 Appendix — (PDF) [file pone.0119986.s004.pdf]

S4 Appendix: Factor, spatial scales, and models considered to describe the effects of conspecific neighbors on reproductive output

Table S4A. Factors considered when modeling the effects of presence and abundance of conspecifics on reproductive output of Ferruginous pygmy-owls in northern Sonora, Mexico, 2001-2010. For presence and number of neighbors we considered a maximum distance of 1.5 km from focal nests because preliminary analyses indicated little effect beyond this distance.

Variable	Scale	Abbreviation	Definition	Units
Total Occupancy	Population	Occ_{total}	Proportion of territory patches occupied across the entire study in each year	Proportion
Regional Occupancy	Region	Occ_{region}	Proportion of territory patches occupied within each watershed region in each year	Proportion
Presence of neighbor	Local	PresN	Presence or absence of nearest neighbor nesting pair within 1.5 km of focal site	0 or >0 individuals
Number of neighbors	Local	No.pres	Number of nearest neighbor nesting pairs within 1.5 km of focal site	0, 1, or 2 individuals
Density	Local	Density	Number of nearest neighbor nesting pairs per km around focal site (see text)	no./km^2

Table S4B. Rankings and estimated slope parameters for 5 hypothesized models that explained the effects of presence and abundance of conspecifics on reproductive output of ferruginous pygmy-owls in northern Sonora Mexico, 2001-2010.

Factor - Scale	Formula	K	LL	ΔAICc	w_i	Slope ± SE
Density - Local	Density	4	-920.79	0.00	0.379	$\beta_1 = -0.18 \pm 0.084$
Number of Neighbors - Local	No.pres	4	-921.29	1.01	0.229	$\beta_1 = -0.31 \pm 0.17$
Presence of Neighbor - Local	PresN	4	-921.53	1.47	0.181	$\beta_1 = -0.24 \pm 0.13$
Intercepts only	β_0	3	-923.08	2.54	0.106	
Occupancy - Region	Occ_{region}	4	-922.56	3.54	0.065	$\beta_1 = 0.42 \pm 0.41$
Occupancy - Population	Occ_{total}	4	-923.06	4.54	0.039	$\beta_1 = 0.12 \pm 0.61$
