# Supplementary material for: Spatial, Temporal, and Density-Dependent Components of Habitat Quality for a Desert Owl
Source: PLoS One. 2015 Mar 18;10(3):e0119986. doi: 10.1371/journal.pone.0119986 (PMC4364994; doi:10.1371/journal.pone.0119986)
Supplement: S5 Appendix — (PDF) [file pone.0119986.s005.pdf]

S5 Appendix: Models and estimates of the interactive effects of spatial, temporal, and conspecific factors on reproductive output of ferruginous pygmy-owls in northwest Mexico, 2001-2010.

Table S5A. Rankings of models that described the individual, additive, and interactive effects of spatial and temporal factors and conspecific density on reproductive output of ferruginous pygmy-owls in northwest Mexico, 2001-2010. Factors included in models were identified by assessing a range of a priori hypotheses and are the same factors included in the best approximating models for each component.

Hypothesis	Model	K	LL	ΔAICc	w_i
Habitat × Weather + Density	lnCav + Comm + Hab_f + lnCav*Hab_f + $Frag_{hab}$ + lnT_{brood} + lnP_{yr} + lnT_{brood}*lnP_{yr} + $NDVI_{yr}^2$ + Density + lnP_{yr}*Hab_f + lnT_{brood}*Hab_f + lnT_{brood}*lnP_{yr}*Hab_f	16	-882.99	0.00	0.264
Habitat + Weather × Density	lnCav + Comm + Hab_f + lnCav*Hab_f + $Frag_{hab}$ + lnT_{brood} + lnP_{yr} + lnT_{brood}*lnP_{yr} + $NDVI_{yr}^2$ + Density + lnP_{yr}*Density	14	-885.20	0.15	0.245
Habitat × Weather × Density	lnCav + Comm + Hab_f + lnCav*Hab_f + $Frag_{hab}$ + lnT_{brood} + lnP_{yr} + lnT_{brood}*lnP_{yr} + $NDVIyr^2$ + Density + $Frag_{hab}$*Density + $Frag_{hab}$*lnP_{yr} + Density*lnP_{yr} + $Frag_{hab}$*Density*lnP_{yr} + Hab_f*Density + Hab_f*$NDVI_{yr}^2$ + Density*$NDVI_{yr}^2$ + Hab_f*Density*$NDVI_{yr}^2$	21	-877.83	0.55	0.201
Habitat + Weather + Density	lnCav + Comm + Hab_f + lnCav*Hab_f + $Frag_{hab}$ + lnT_{brood} + lnP_{yr} + lnT_{brood}*lnP_{yr} + $NDVI_{yr}^2$ + Density	13	-886.80	1.22	0.144
Habitat × Density + Weather	lnCav + Comm + Hab_f + lnCav*Hab_f + $Frag_{hab}$ + lnT_{brood} + lnP_{yr} + lnT_{brood}*lnP_{yr} + $NDVI_{yr}^2$ + Density + Hab_f*Density + $Frag_{hab}$*Density + Comm*Density	16	-884.41	2.84	0.064

Habitat × Weather	$lnCav + Comm + Hab_f + lnCav*Hab_f + Frag_{hab} + lnT_{brood} + lnP_{yr} + lnT_{brood}*lnP_{yr} + NDVI_{yr}^2 + lnP_{yr}*Hab_f + lnT_{brood}*Hab_f + lnT_{brood}*lnP_{yr}*Hab_f$	15	-885.76	3.40	0.048
Habitat + Weather	$lnCav + Comm + Hab_f + lnCav*Hab_f + Frag_{hab} + lnT_{brood} + lnP_{yr} + lnT_{brood}*lnP_{yr} + NDVI_{yr}^2$	12	-889.43	4.36	0.030
Habitat × Density	$lnCav + Comm + Hab_f + lnCav*Hab_f + Frag_{hab} + Density + Hab_f*Density + Frag_{hab}*Density + Comm*Density$	12	-892.39	10.29	0.002
Habitat + Density	$lnCav + Comm + Hab_f + lnCav*Hab_f + Frag_{hab} + Density$	9	-895.75	10.72	0.001
Habitat only	$lnCav + Comm + Hab_f + lnCav*Hab_f + Frag_{hab}$	8	-896.87	10.87	0.001
Weather × Density	$lnT_{brood} + lnP_{yr} + lnT_{brood}*lnP_{yr} + NDVI_{yr}^2 + lnP_{yr}*Density$	9	-908.67	36.56	0.001
Weather + Density	$lnT_{brood} + lnP_{yr} + lnT_{brood}*lnP_{yr} + NDVI_{yr}^2 + Density$	8	-910.41	37.95	0.001
Weather only	$lnT_{brood} + lnP_{yr} + lnT_{brood}*lnP_{yr} + NDVI_{yr}^2$	7	-915.10	45.27	0.001
Density only	Density	4	-920.79	50.49	0.001

Table S5B. Estimates of slope parameters in models that described the individual, additive, and interactive effects of spatial and temporal factors and conspecific density on reproductive output of ferruginous pygmy-owls in northern Sonora, Mexico 2001-2010. Model rankings are presented in Table 6. Only estimates for models within 5 ΔAICc points are reported

Model (ΔAICc)<br>Factor	β	SE
Habitat × Weather + Density (0.00)		
lnCav	0.22	0.20
Comm(SDG)	0.33	0.18
Hab_f	-17.8	6.6
lnCav*Hab_f	0.019	0.010
$Frag_{hab}$	-0.18	0.084
lnT_{brood}	-135.9	39.3
lnP_{yr}	-133.8	40.0
lnT_{brood}*lnP_{yr}	37.0	11.0
$NDVI_{yr}^2$	21.9	8.9
Density	-0.19	0.080
lnP_{yr}*Hab_f	4.90	1.83
lnT_{brood}*Hab_f	4.87	1.80
lnT_{brood}*lnP_{yr}*Hab_f	-1.35	0.50
Habitat + Weather × Density (0.15)		
lnCav	0.22	0.20
Comm(SDG)	0.34	0.18
Hab_f	-0.045	0.034
lnCav*Hab_f	0.016	0.010

$Frag_{hab}$	-0.19	0.084
lnT_{brood}	-33.6	13.7
lnP_{yr}	-30.4	13.2
$lnT_{brood}*lnP_{yr}$	8.51	3.64
$NDVI_{yr}^2$	23.0	8.82
Density	0.96	0.65
Density*lnP_{yr}	-0.32	0.18
Habitat × Weather × Density (0.55)		
lnCav	0.31	0.21
Comm(SDG)	0.35	0.18
Hab_f	-0.011	0.039
lnCav*Hab_f	0.012	0.011
$Frag_{hab}$	-0.86	0.74
lnT_{brood}	-36.1	13.9
lnP_{yr}	-33.3	13.5
$lnT_{brood}*lnP_{yr}$	9.24	3.71
$NDVI_{yr}^2$	38.3	21.5
Density	1.76	1.53
Density*$Frag_{hab}$	0.087	0.842
$Frag_{hab}*lnP_{yr}$	0.23	0.21
Density*lnP_{yr}	-0.29	0.41
Density*Hab_f	-0.038	0.020
$Hab_f*NDVI_{yr}^2$	-1.89	1.25
Density*$NDVI_{yr}^2$	-22.8	29.9
Density*$Frag_{hab}*lnP_{yr}$	-0.093	0.23
Density*$Hab_f*NDVI_{yr}^2$	2.88	1.71
Habitat + Weather + Density (1.22)		
lnCav	0.23	0.20

Comm(SDG)	0.34	0.18
Hab_f	-0.043	0.034
$lnCav*Hab_f$	0.017	0.010
$Frag_{hab}$	-0.18	0.084
lnT_{brood}	-35.6	13.7
lnP_{yr}	-32.3	13.2
$lnT_{brood}*lnP_{yr}$	8.99	3.64
$NDVI_{yr}^2$	23.0	8.8
Density	-0.182	0.080
Habitat × Density + Weather (2.84)		
lnCav	0.30	0.21
Comm(SDG)	0.44	0.21
Hab_f	-0.021	0.038
$lnCav*Hab_f$	0.013	0.011
$Frag_{hab}$	-0.078	0.10
lnT_{brood}	-34.9	13.7
lnP_{yr}	-31.8	13.2
$lnT_{brood}*lnP_{yr}$	8.84	3.64
$NDVI_{yr}^2$	21.5	8.89
Density	0.69	0.43
Hab_f*Density	-0.025	0.016
$Frag_{hab}$*Density	-0.21	0.11
Comm*Density	-0.18	0.16
Habitat × Weather (3.4)		
lnCav	0.22	0.20
Comm(SDG)	0.34	0.18
Hab_f	-17.5	6.60
$lnCav*Hab_f$	0.020	0.010

$Frag_{hab}$	-0.19	0.085
lnT_{brood}	-129.7	39.4
lnP_{yr}	-127.4	40.1
$lnT_{brood}*lnP_{yr}$	35.2	11.0
$NDVI_{yr2}$	21.9	8.91
$lnP_{yr}*Hab_f$	4.79	1.83
$lnT_{brood}*Hab_f$	4.80	1.81
$lnT_{brood}*ln_{Pyr}*Hab_f$	-1.32	0.50
Habitat + Weather (4.36)		
lnCav	0.24	0.20
Comm(SDG)	0.36	0.18
Hab_f	-0.047	0.034
$lnCav*Hab_f$	0.018	0.010
$Frag_{hab}$	-0.19	0.085
lnT_{brood}	-31.5	13.6
lnP_{yr}	-28.8	13.2
$lnT_{brood}*lnP_{yr}$	8.01	3.63
$NDVI_{yr}^2$	23.0	8.89
